# Buprenorphine extended-release (Ethiqa XR) impacts the immunological response in mice exposed to aerosolized *Burkholderia pseudomallei* or *Yersinia pestis*

**DOI:** 10.3389/fimmu.2026.1823747

**Published:** 2026-05-13

**Authors:** Carlos I. Rodriguez, Christopher P. Klimko, Michael L. Davies, Jennifer L. Dankmeyer, Nathaniel O. Rill, Melissa Hunter, Brian A. Smith, Taloria K. Wheeler, Wendy M. Webster-Zahnow, Andre M. Swamotz, Yunuen Hernandez-Viezcas, Christian Xander, Elsie E. Martinez, Ronald G. Toothman, Kevin D. Mlynek, Joel A. Bozue, Ju Qiu, Sergei S. Biryukov, Christopher K. Cote

**Affiliations:** 1 Bacteriology Division, United States Army Medical Research Institute of Infectious Diseases (USAMRIID), Frederick, MD, United States; 2 Biostatistics Division, United States Army Medical Research Institute of Infectious Diseases (USAMRIID), Frederick, MD, United States

**Keywords:** analgesia, bacteria, immunology, inflammation, melioidosis, mice, plague

## Abstract

**Introduction:**

Ethiqa XR is an extended-release formulation of the potent partial opioid buprenorphine and has been FDA-indexed for mice and other laboratory animal species to relieve pain. Unfortunately, the use of analgesia may produce confounding effects that distort physiological and pathophysiological responses during laboratory animal studies. Since various reports have indicated that Ethiqa XR can affect the inflammatory response in several *in vivo* models, we sought to evaluate the effects of Ethiqa XR treatment on the immune response to and disease pathogenesis associated with bacterial biothreat agents.

**Methods:**

BALB/c and C57BL/6 mouse strains were treated or not with Ethiqa XR before and 48 h after challenge with aerosolized *Burkholderia pseudomallei* K96243 or *Yersinia pestis* CO92. Control mice were similarly treated with Ethiqa XR but were not challenged. Mice were euthanized 60-72 hours post-treatment/challenge to harvest blood, serum, lung, brain and spleen for bacterial burden and immunological profiling.

**Results:**

Both C57BL/6 and BALB/c mouse strains showed higher bacterial dissemination to the spleen 60-72 h after *B. pseudomallei* challenge when treated with Ethiqa XR. Consistently, we found increased concentration of pro-inflammatory cytokines in the spleen (e.g., IFN-γ and IL-6) in Ethiqa XR-treated mice. Compared to BALB/c, C57BL/6 mice had trends of higher cytokine dysregulation not only in the spleens, but also in the lungs and brains. In contrast, we found less profound differences in either mouse strain treated with Ethiqa XR and challenged with *Y. pestis*. In the absence of bacterial challenge, both BALB/c and C57BL/6 mouse strains treated with Ethiqa XR had an overall higher concentration of pro-inflammatory cytokines in the lungs and spleens. However, C57BL/6 mice showed a higher dysregulation of the cytokine profile in both lungs and spleens. We also found decreased macrophage activity (iNOS concentration) in the spleens of BALB/c mice and decreased neutrophil activity (MPO concentration) in the lung of C57BL/6 mice treated with Ethiqa XR.

**Discussion:**

These results suggest that the effect of Ethiqa XR on the immune response and disease pathogenesis increases the complexity of data interpretation. Thus, prior to providing analgesia to laboratory animals, bridging studies and cost benefit analyses must be considered to avoid misinterpretation of immunological data collected during the development, testing, and evaluation of medical countermeasures.

## 1 Introduction

A major pillar of laboratory animal care and use is to attempt to limit pain or distress, when possible, without interfering with the scientific objectives of the *in vivo* experiments. However, analgesia can introduce several uncontrolled variables in the experimental design. For example, we previously reported that splenocytes from female C57BL/6 and BALB/c mice vaccinated with a live attenuated *Yersinia pestis* vaccine and treated with acetaminophen showed a reduced IFN-γ recall response (1). Moreover, acetaminophen-treated female C57BL/6 mice had lower IgG2c/IgG1 ratios, while the IgG2a/IgG1 ratio in female BALB/c mice was reduced after either acetaminophen or meloxicam treatment (1). However, outside of vaccine development research, very little data exist on the impacts of analgesia on disease pathogenesis.

Buprenorphine is a potent partial opioid approved by the U.S. Food and Drug Administration (FDA) (2). Ethiqa XR (buprenorphine extended-release injectable suspension) is bound in a lipid capsule and suspended in a medium chain fatty acid triglyceride (3). It has been FDA-indexed for mice and other animal species to effectively relieve pain for extended periods. While the use of Ethiqa XR may limit the pain and distress experienced by laboratory animals, several reports have shown contradictory effects on the inflammatory responses following treatment. For example, acute or chronic treatment with buprenorphine did not affect lymphoproliferation, natural killer (NK) cell activity, IL-2, or interferon-γ production in male Swiss mice (4). In the context of vaccination, a recent study showed that treatment with buprenorphine, and other analgesics such as acetaminophen and meloxicam, did not alter the antibody responses of male C57BL/6J mice after primary or repeated subcutaneous immunization with a dose of recombinant protective antigen (PA) from *Bacillus anthracis*, with complete Freund’s adjuvant (CFA) or incomplete Freund’s adjuvant (IFA) (5). Another study reported that treatment with buprenorphine (either normal or sustained-release formulations) did not significantly change the antibody response of ovalbumin + CFA-immunized female CD1:Crl mice. However, the traditional formulation of buprenorphine seemed to reduce the secretion of IL-10, TNF-α, and IFN-γ by ovalbumin-stimulated splenocytes; whereas, the sustained-release buprenorphine did not have a profound effect, except for an increase of IL-10, when compared to saline-treated group (6). It is worth noting that mice were also given the vehicle for the sustained-release buprenorphine on its own, and it did not affect the concentration of these cytokines (6).

In the context of sepsis, two high doses of buprenorphine before and after cecal ligation and perforation (CLP), did not significantly affect the survival of female C57BL/6 mice up to 20 days compared to saline treated controls (7). However, this was accompanied by a transient decrease of total plasma white blood cells and lymphocytes at 24 h that normalized at 48 h (7). In contrast, a significant reduction in survival was reported for male mice, suggesting a possible gender-specific effect for buprenorphine (7). However, in both male and female mice, no significant differences were found in IL-6, IL-10, KC, MIP2-α, and IL-1β cytokine responses relative to the control (7). Interestingly, when the control groups were compared, male mice had better survival (~70%) than female mice (~40%) after CLP (7). In the context of sterile inflammation, in a collagen-induced arthritis (CIA) model, the serum concentration of IFN-γ was significantly decreased in male DBA/1J mice treated with buprenorphine. Moreover, although not significant, buprenorphine treatment decreased the concentration of IL-2, IL-6, and TNF-α, suggesting a protective effect against CIA (8). These prior studies suggest differences related to mouse strain and sex in response to buprenorphine treatment.

In an attempt to include analgesia in our laboratory studies, we characterized some of these potential alterations to the immune response and disease pathogenesis using two different mouse models of disease following exposure to aerosolized biothreat agents *Y. pestis* and *B. pseudomallei*. Negative control animals in both disease models uniformly succumb to disease or are euthanized in accordance with early endpoint euthanasia criteria and thus, are prime candidates for pain management intervention associated with infection. However, these control animals are essential to use as comparators to assess the immune response in animals that have received medical countermeasures (e.g., vaccines, immune modulators, or therapeutics). Accordingly, we cannot discount the impact of analgesics on the immune response of these control animals and their influence on downstream data analyses and interpretations when developing or evaluating novel medical countermeasures.

## 2 Materials and methods

### 2.1 Animal research

All animal research was conducted under an animal use protocol approved by the USAMRIID Institutional Animal Care and Use Committee (IACUC) in compliance with the Animal Welfare Act, Public Health Service Policy, and other federal statutes and regulations relating to animals and experiments involving animals. The facility where this research was conducted is accredited by the AAALAC International and adheres to the principles stated in The Guide for the Care and Use of Laboratory Animals (National Research Council, 2011).

### 2.2 Bacteria preparation

*Y. pestis* CO92 preparation for aerosol challenge involved growing bacteria on tryptose blood agar (TBA) (Becton Dickinson, Franklin Lakes, NJ), base slants for approximately 48 h at 28-30 °C. Bacteria from Difco™ TBA slants were then suspended in Difco™ heart infusion broth (Becton Dickinson) medium supplemented with 0.2% xylose (Sigma Aldrich, St. Louis, MO) (HIBX) to an initial OD_620_ of approximately 0.01 and incubated for approximately 24 h at 28–30 °C. Then, the cultures were harvested by centrifugation (10 min at 13,000 x g) and suspended in heart infusion broth (HIB) medium (no xylose) to the concentration yielding the determined number of LD_50_ doses for each mouse strain. A solution of 10 mM potassium phosphate, pH 7.3–7.4 (KPhos) was used to dilute bacterial inocula. Challenge doses were determined by serial dilutions in KPhos buffer and plating on SBA.

*B. pseudomallei* K96243 cultures were started from a freezer stock and grown for approximately 16 h (until approximately late log phase) in Gibco™ Tryptone broth (Thermo Fisher) with 4% glycerol (Sigma Aldrich) and 5% NaCl (Sigma Aldrich)(GTB) at 37 °C at 150 RPM in an orbital shaker. The optical density was determined at OD_620_, the culture was centrifuged for 10 min at 4,648 x g and then resuspended in fresh GTB. The OD_620_ was determined again, and the culture was diluted to the desired concentration yielding the determined number of LD_50_ doses for each mouse strain. Challenge doses were determined by serial dilutions in PBS buffer and plating on SBA.

### 2.3 Challenge and treatment conditions

**Figure 1:**
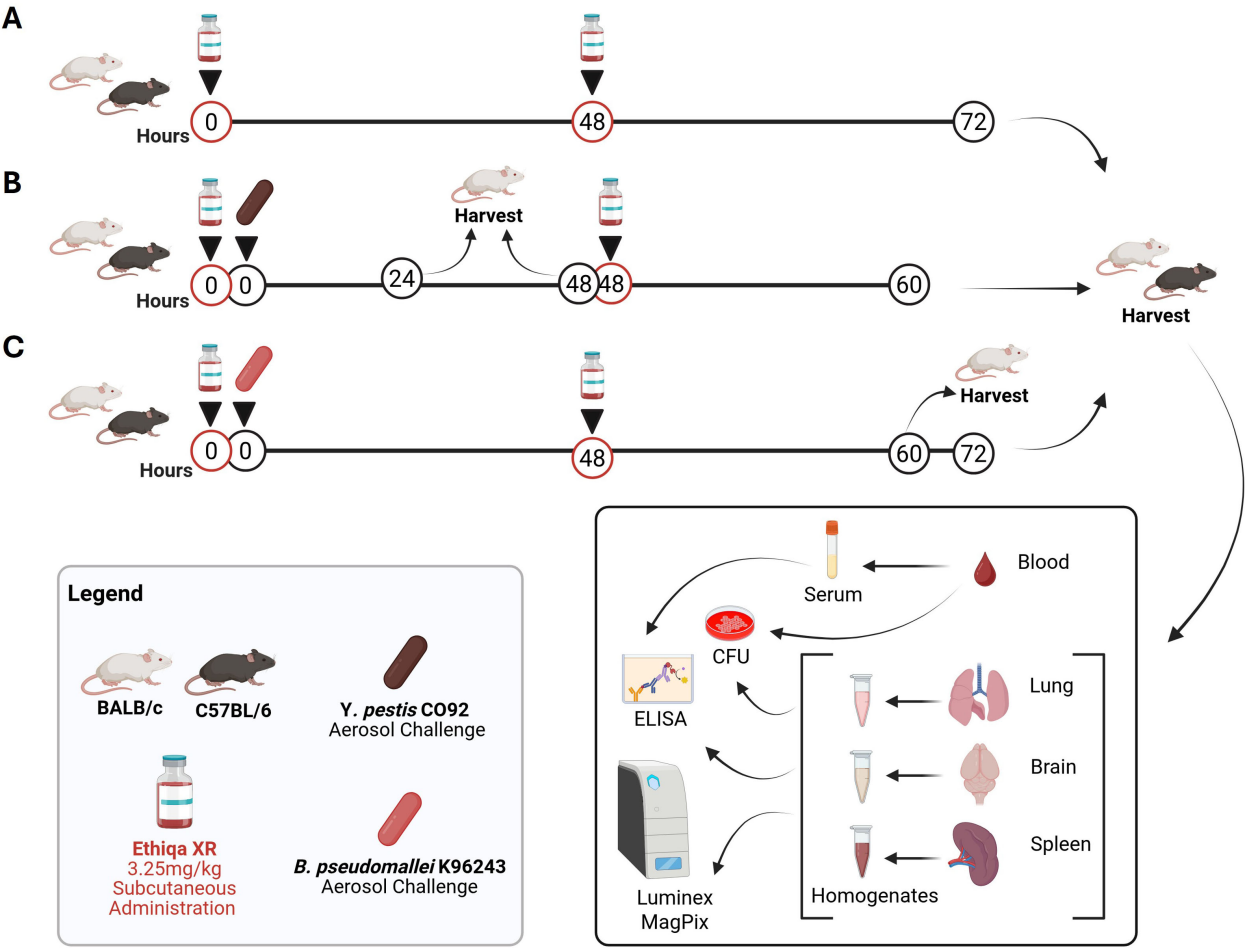
Experimental design. BALB/c and C57BL/6 female mice were subcutaneously treated with 3.25 mg/kg Ethiqa XR at 0 h and 48 h. Control mice were not treated. **(A)** At 72 h, mice were euthanized to harvest tissues (*n* = 5 mice per group). **(B)** BALB/c (*n* = 23 per group) and C57BL/6 (*n* = 5 per group) were treated with Ethiqa XR or not (control) followed by whole body challenge with aerosolized *Y. pestis* CO92. At 24 h and 48 h post-challenge, treated and control BALB/c mice were euthanized to harvest tissues (*n* = 7 per group) and at 48 h, another set of BALB/c mice (*n* = 7 per group) received an additional dose of Ethiqa XR. At 60 h, both strains of mice were euthanized to harvest tissues. **(C)** BALB/c (*n* = 20 per group) and C57BL/6 *(n* = 10 per group) were treated with Ethiqa XR or not (control) followed by whole body challenge with aerosolized *B. pseudomallei* K96243. At 48 h, mice were treated with Ethiqa XR or not (control). At 60 h post-challenge, a set of control or treated (*n* = 5 per group) BALB/c mice were euthanized to harvest tissues. All other animals were euthanized to harvest tissues at 72 h (Note: *n* = 4 per group for BALB/c as one animal per group succumbed to disease).

The challenge and treatment conditions are depicted in **Figure 1**. Briefly, 6-8-week-old female BALB/c and C57BL/6 mice were acquired from Charles River (Frederick, MD) and allowed to acclimate for at least 5 days prior to manipulations. Mice were randomly assigned to groups prior to treatment; however, the study was not blinded. Mice (7–9 weeks at time of treatment) were subcutaneously treated with 3.25 mg/kg Ethiqa XR. Non-treated mice were used as control. For exposure to aerosolized bacteria, mice were transferred to wire mesh cages and transferred to a whole-body aerosol chamber within a class three biological safety cabinet located inside a BSL-3 laboratory. Mice were then exposed to aerosols of *Y. pestis* CO92 or *B. pseudomallei* K96243 suspension created by a three-jet Collison nebulizer (9). Samples were collected from an all-glass impinger (AGI) vessel and actual delivery dose of bacteria was determined as the number of colony forming units (CFU) by plating on sheep blood agar (SBA) plates. At 48 h post-challenge, mice were again treated with 3.25mg/kg Ethiqa XR subcutaneously. For *Y. pestis* CO92, the actual inhaled dose was calculated to be 4.42x10^5^ CFU (~6.5 LD_50_) for BALB/c and 7.37X10^5^ CFU (~25.4 LD_50_) for C57BL/6 mice (10) (data not shown). For *B. pseudomallei* K96243, the actual inhaled doses for BALB/c and C57BL/6 were 4.59x10^2^ CFU (~18.4 LD_50_) and 2.66x10^3^ CFU (~7.4 LD_50_), respectively (9).

### 2.4 Bacteriology and organ processing

The tissues harvested from necropsied mice, included lungs, spleens, brains, and blood as shown in **Figure 1**. Mice, anesthetized with approximately 0.2 mL/20 g of body weight of a mixture of ketamine (10 mg/mL)-acepromazine (1 mg/mL)-xylazine (2 mg/mL), underwent a terminal blood collection via the axillary vessels, and then were euthanized by cervical dislocation prior to organ harvesting. Serum extraction was performed using BD Microtainer^®^ blood collection tubes (Becton Dickinson, Franklin Lakes, NJ). The organs were weighed, homogenized in 1 mL PBS per sample with disposable PRECISION™ homogenizers (Covidien, Dublin, Ireland), and the CFU in the homogenate were quantified by plating on SBA plates. Undiluted homogenate and ten-fold dilutions in KPhos were plated in duplicate. The limit of detection (LOD) was approximately 100 CFU/mL blood or 5 CFU/organ. After CFU determinations, samples were radiation-inactivated, sterility checked and stored at -80 °C for immunological analyses.

### 2.5 ELISAs

The concentration of inducible nitric oxide synthase (iNOS) and eosinophil peroxidase (EPO) in tissue homogenate supernatants were quantified using Cusabio (Houston, TX) ELISA kits following manufacturer recommendations. Similarly, to quantify the concentration of myeloxidase (MPO), we employed an ELISA kit from Hycult Biotech (East Hartford, CT). The quantification of complement C3a and C5b-9 in the serum was performed using Novus Biologicals (Centennial, CO) and LSBio (Seattle, WA) kits, respectively. Values below the lowest limit of detection were imputed with that lowest value of the standard curve before accounting for the dilution factor of samples.

### 2.6 Luminex cytokine analysis

Tissue homogenate supernatants were measured for cytokine expression levels using the ProcartaPlex Mouse Cytokine & Chemokine panel 1A, 36-plex (Thermo Fisher, Bothell, WA) and MagPix instrument per manufacturer’s instructions. Cytokine results that were above or below the highest or lowest values of the standard curve were imputed to the value of the highest or lowest standard. In addition, any cytokine whose standard curve had low R^2^ value (< 0.95) or a bead count of < 35 was removed from analysis.

### 2.7 Statistical analysis

The Luminex and ELISA data are shown as mean of the log-transformed concentration values ± SD or the value with geometric mean bar. Pairwise treatment groups were compared by linear mixed effects model. No multiplicity adjustment was applied. Analysis was implemented in SAS version 9.4 (SAS Institute Inc., Carry, NC). Bacterial burden was analyzed with Prism GraphPad Prism software perming unpaired nonparametric Mann-Whitney tests (0.05 = threshold for *p* value rank comparisons).

## 3 Results

### 3.1 BALB/c and C57BL/6 mice treated with Ethiqa XR show an overall pro-inflammatory profile in the lung and spleen

**Figure 2:**
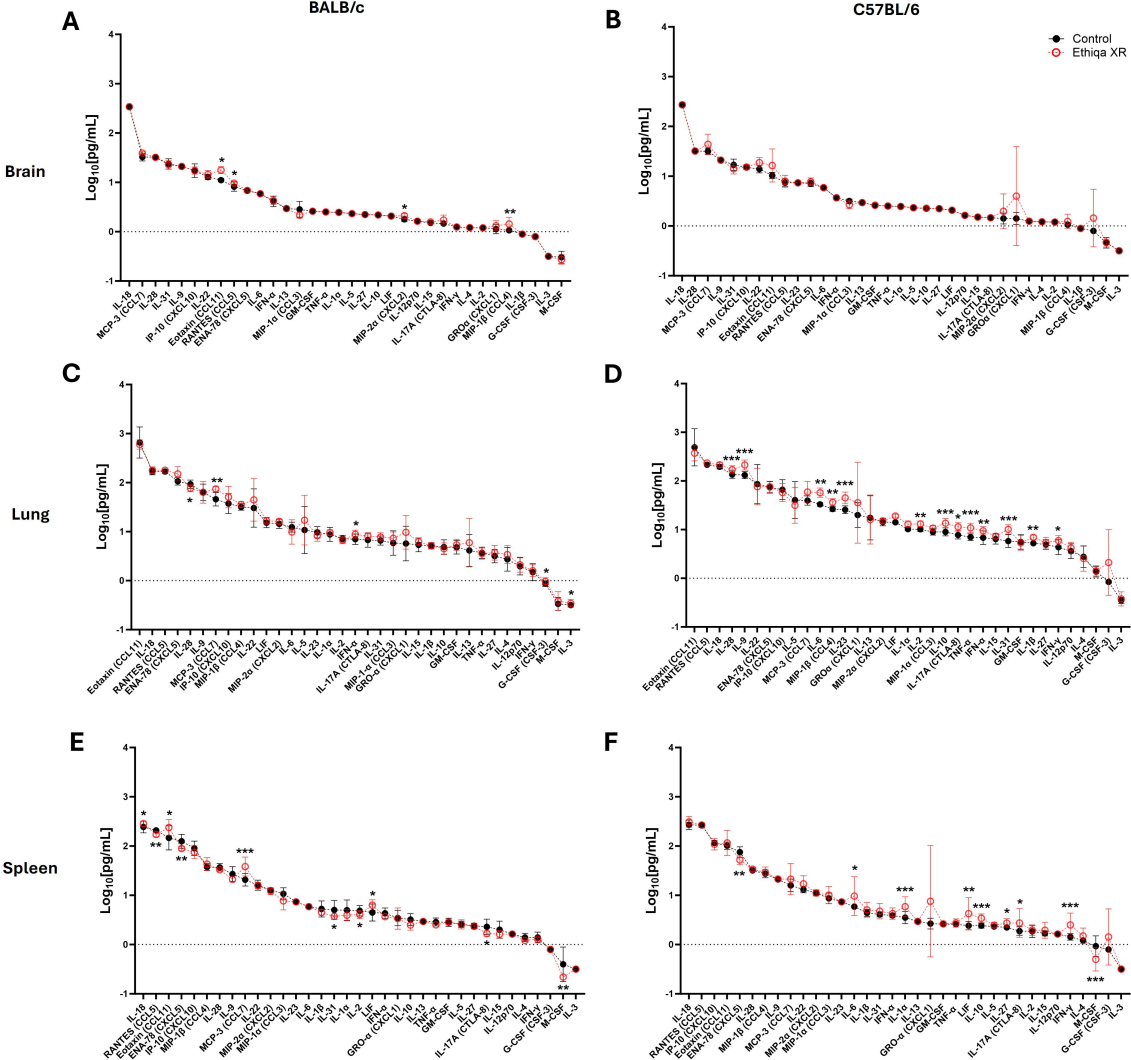
Ethiqa XR treatment promotes a more pronounced pro-inflammatory response in C57BL/6 mice. The concentration of cytokine in the **(A, B)** brain, **(C, D)** lung, and **(E, F)** spleen homogenates from BALB/c (*n* = 5 per group) and C57BL/6 (*n* = 5 per group) mice treated with Ethiqa XR and control. Data (mean ± SD) show log-transformed concentration values. Pairwise treatment groups were compared by linear mixed effects model with no multiplicity adjustment applied. Statistically significant changes are marked as: * (*p* ≤ 0.05), ** (*p* ≤ 0.005), *** (*p* ≤ 0.0005).

Opioid use alters the function of the central nervous system, specifically the brain. Since buprenorphine (the active ingredient of Ethiqa XR) is an opioid analgesic, we initially investigated the effect of Ethiqa XR on the inflammatory profile in brain homogenates from BALB/c and C57BL/6 mice, as described in **Figure 1A**. After treatment with Ethiqa XR, BALB/c mice had significantly higher levels of Eotaxin (CCL11), RANTES (CCL5), MIP-2α (CXCL2), and MIP-1β (CCL4) in brain tissue (**Figure 2A**). No significant differences were observed in the brain from Ethiqa XR-treated compared to non-treated C57BL/6 mice (control) (**Figure 2B**). Since buprenorphine has some respiratory depression effects (11), the cytokine response in lung homogenates was also evaluated. BALB/c mice did not show a substantial change in the cytokine response after treatment with Ethiqa XR (**Figure 2C**). Compared to control mice, five cytokines produced statistically significant differences in BALB/c mice: MCP-3 (CCL7), IFN-α, G-CSF (CSF-3), and IL-3 were higher, and IL-28 was lower (**Figure 2C**). In contrast, lung homogenates from C57BL/6 mice treated with Ethiqa XR showed higher dysregulation, as more cytokines (13/35) were significantly upregulated, compared to control mice: IL-28, IL-9, IL-6, MIP-1β (CCL4), IL-23, IL-2, IL-10, IL-17A (CTLA-8), TNF-α, IFN-α, IL-31, IL-1β, and IFN-γ (**Figure 2D**). Next, the cytokine levels in spleens were evaluated to further understand the effects of Ethiqa XR on the inflammatory responses of BALB/c and C57BL/6 mice (**Figures 2E, F**). Spleen homogenates from Ethiqa XR-treated BALB/c had significantly higher levels of IL-18, Eotaxin (CCL11), MCP-3 (CCL7), and LIF but lower RANTES (CCL5), ENA-78 (CXCL10), IL-31, IL-17A (CTLA-8), IL-2, and M-CSF (**Figure 2E**). Compared to the control, spleen homogenates from Ethiqa XR-treated C57BL/6 mice had statistically higher levels of IL-6, IL-1α, LIF, IL-10, IL-27, IL-17A (CTLA-8), and IFN-γ and lower ENA-78 (CXCL10) and M-CSF (**Figure 2F**). These data suggest that treatment with Ethiqa XR promotes an overall pro-inflammatory response and that C57BL/6 mice are more susceptible than BALB/c mice to Ethiqa XR-induced immune modulation.

**Figure 3:**
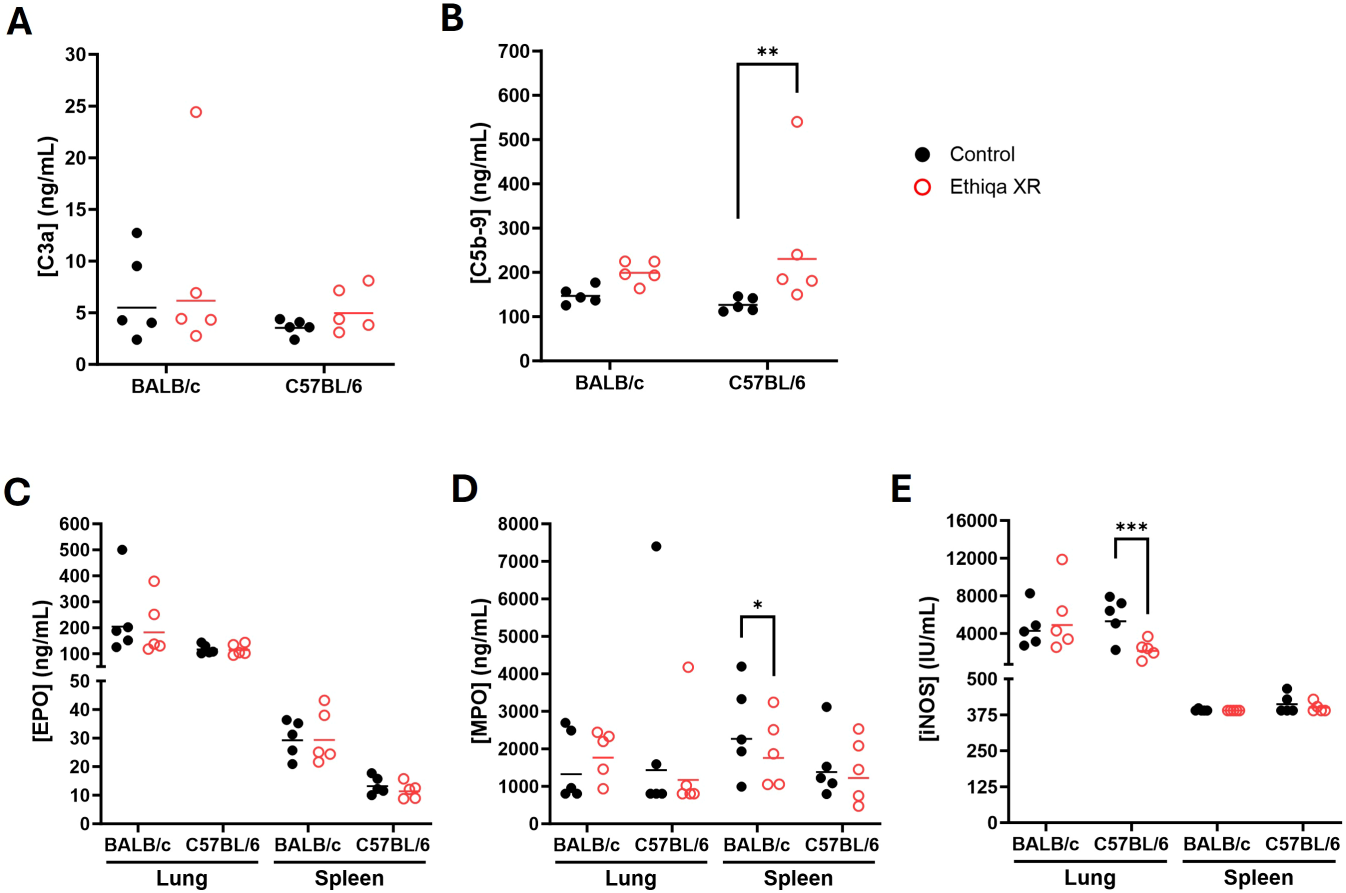
Disruption in membrane attack complex, macrophage, and neutrophil activity following Ethiqa XR treatment. Complement activity was measured in the serum of **(A)** C3a and **(B)** C5b-9 from Ethiqa XR-treated and control BALB/c (*n* = 5 per group) and C57BL/6 (*n* = 5 per group) mice. Analysis of eosinophil, macrophage, and neutrophil activity by measuring the concentration of **(C)** eosinophil peroxidase (EPO), **(D)** myeloxidase (MPO), and **(E)** inducible nitric oxide synthase (iNOS) from the specified tissue homogenates from Ethiqa XR-treated and control BALB/c (*n* = 5 per group) and C57BL/6 (*n* = 5 per group). Horizontal bars show the geometric mean. Pairwise treatment groups were compared by linear mixed effects model. Statistically significant changes are marked as: * (*p* ≤ 0.05), ** (*p* ≤ 0.005), *** (*p* ≤ 0.0005).

These pro-inflammatory responses in lung and spleen, prompted us to investigate the status of complement activation (**Figures 3A, B**). Excessive anaphylatoxin (e.g., C3a and C5a) and membrane attack complex (C5b-9) production is associated with increased inflammation and lung damage. No significant changes in the serum levels of C3a were detected in either strain (**Figure 3A**). Interestingly, we found elevated C5b-9 levels in the serum of both BALB/c and C57BL/6 mice treated with Ethiqa XR, although only statistically significant for C57BL/6 (**Figure 3B**). Next, we measured the levels of Eosinophil Peroxidase (EPO), Myeloperoxidase (MPO), and Inducible nitric oxide synthase (iNOS) to assess eosinophil, neutrophil, and macrophage activity, respectively, in the lung and spleen (**Figures 3C–E**). As expected, there was more eosinophil activity in the lung compared to the spleen for both strains (**Figure 3C**). While the magnitude of the activity was similar in the lungs for both strains with any treatment, BALB/c mice showed a higher baseline (control values) activity in the spleen than C57BL/6 (**Figure 3C**). Treatment with Ethiqa XR did not significantly affect the EPO levels in the lung or spleen of BALB/c and C57BL/6 mice (**Figure 3C**). BALB/c mice treated with Ethiqa XR had a slightly elevated MPO concentration (neutrophil activity) in the lungs but a statistically lower concentration in the spleen, relative to control (**Figure 3D**). No significant changes were detected in C57BL/6 mice (**Figure 3D**). The macrophage activity (iNOS levels), similar to eosinophils (EPO), was higher in the lungs than in the spleen for both strains (**Figure 3E**). However, C57BL/6 mice treated with Ethiqa XR showed lower levels of iNOS in the lung when compared to control, whereas no changes were detected for BALB/c mice (**Figure 3E**). In the spleen, the iNOS levels of all mice were close to or below the lowest limit of detection (**Figure 3E**).

### 3.2 BALB/c and C57BL/6 mice challenged with aerosolized *Y. pestis* show increased neutrophil and complement activity but no cytokine response alterations when treated with Ethiqa XR

We sought to investigate whether Ethiqa XR has any effect on mice challenged with aerosolized *Y. pestis* CO92. For this, female BALB/c or C57BL/6 mice were treated with 3.25 mg/kg Ethiqa XR immediately before and approximately 48 h after challenge (**Figure 1B**). Blood, lung, brain, and spleen were harvested and processed to determine bacterial burden in each organ as well as to assess the cytokine response in the lung, brain and spleen. It is worth noting that the last time-point was performed earlier than expected (60 h vs 72 h) due to the advanced clinical signs (e.g., labored breathing and decreased normal behaviors) of pneumonic plague in BALB/c mice.

**Figure 4:**
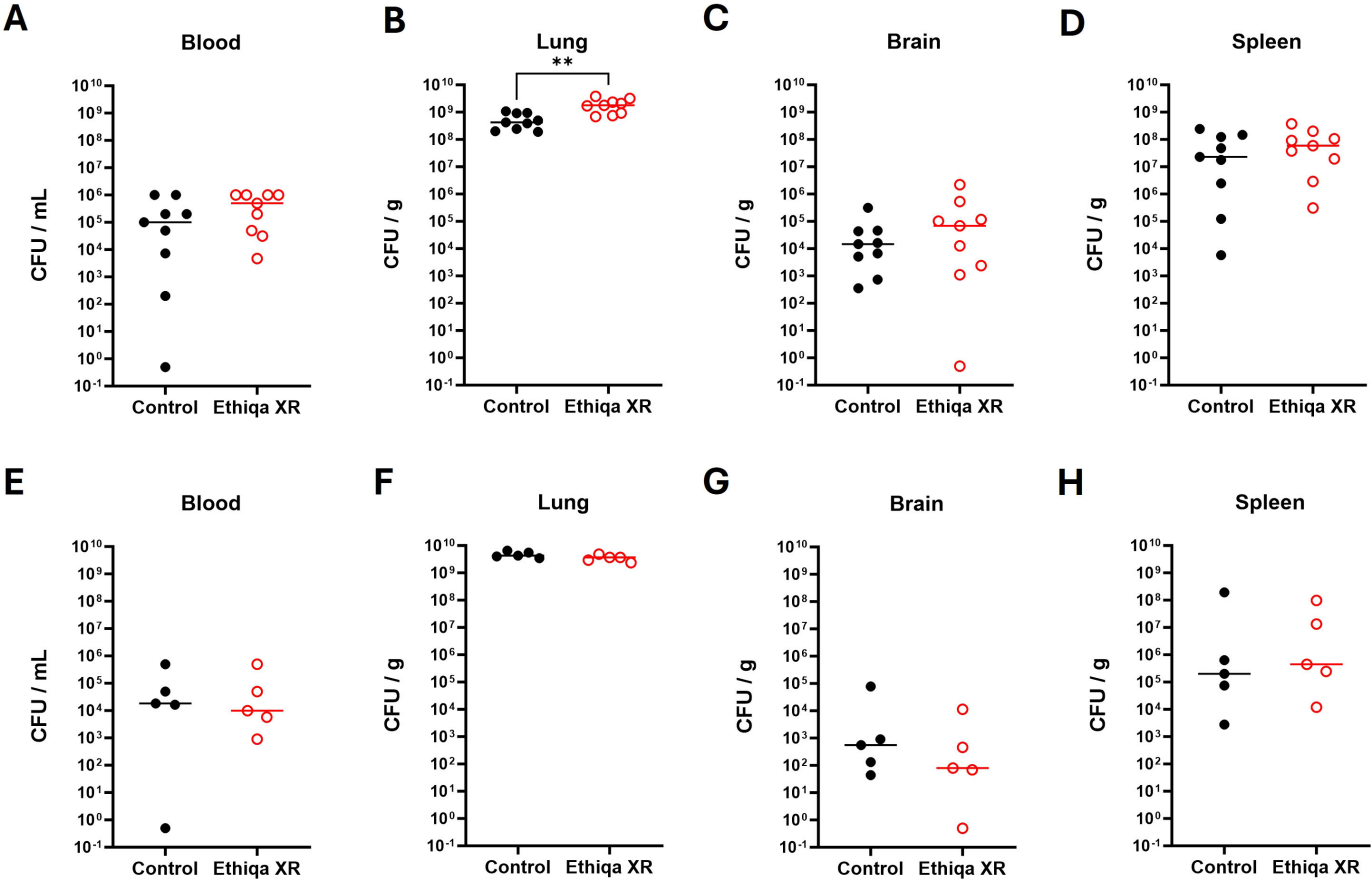
*Y. pestis* bacterial burden in the lungs of BALB/c mice is increased following Ethiqa XR- treatment. The bacterial burden of BALB/c mice in **(A)** blood **(B)** lung, **(C)** brain, and **(D)** spleen homogenates (*n* = 9 per group) 60 h post-challenge with aerosolized *Y. pestis* CO92. The bacterial burden of C57BL/6 mice in **(E)** blood, **(F)** lung, **(G)** brain, and **(H)** spleen homogenates (*n* = 5 per group) 60 h post-challenge with aerosolized *Y. pestis* CO92. Horizontal line shows the median. Statistically significant changes from unpaired nonparametric Mann-Whitney tests are marked as: ** (*p* ≤ 0.005),.

BALB/c mice from control or Ethiqa XR-treated groups were not bacteremic at 24 h, as no CFUs were detected in the blood (**Supplementary Figure 1A**). By 48 h, 100% (7/7) of BALB/c mice from both groups were bacteremic (**Supplementary Figure 1A**). At 60 h, 100% (9/9) of mice from the Ethiqa-treated group were bacteremic but only 88.9% (8/9) of control mice had detectable bacteria in blood (**Figure 4A**). No significant differences were found in the bacterial burden from the lungs at 24 h or 48 h (**Supplementary Figure 1B**). However, at 60 h, lungs from Ethiqa XR-treated mice had significantly more CFU/g compared to the control group (**Figure 4B**). The bacterial burden in the brain and spleen increased over time but no statistical differences were detected between the control and the Ethiqa XR-treated groups (**Supplementary Figures 1C, D**; **Figures 4C, D**).

Since no significant differences in the bacterial burden were observed at 24 h and 48 h between BALB/c groups, for the C57BL/6 strain, we focused our analyses on the 60 h post-challenge time-point (**Figures 4E–H**). Consistent with BALB/c mice, at 60 h we found that 80% (4/5) of C57BL/6 mice from control group were bacteremic whereas 100% (5/5) of Ethiqa-treated mice were bacteremic (**Figure 4E**). We did not find a significant difference in the bacterial burden from the lung, brain, and spleen between Ethiqa XR and control groups (**Figures 4F–H**). Although the bacterial burden in the lung was similar to the BALB/c mice (**Figure 4B** vs **Figure 4F**), the bacterial burden in the brain and the spleen of C57BL/6 was at least 10-fold lower when compared to BALB/c (**Figures 4F–H** vs **Figures 4B–D**).

**Figure 5:**
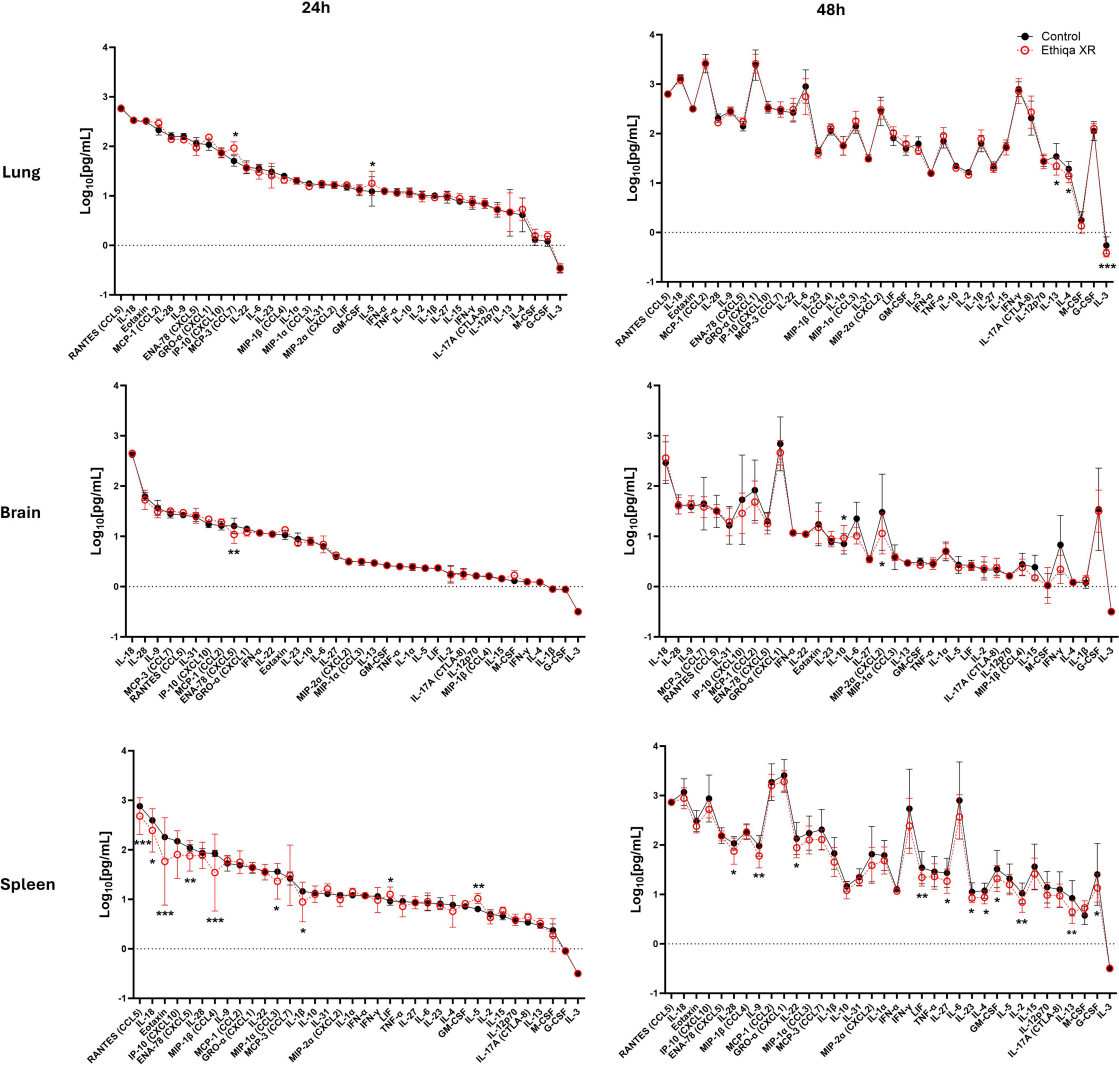
Cytokine response is disrupted following Ethiqa XR treatment in BALB/c mice post *Y. pestis* challenge. Cytokine response in lung, brain, and spleen homogenates from BALB/c mice at 24 h (*n* = 7 per group) and 48 h (*n* = 7 per group) post-challenge with aerosolized *Y. pestis* CO92. Data (mean ± SD) show log-transformed concentration values. Pairwise treatment groups were compared by linear mixed effects model with no multiplicity adjustment applied. Statistically significant changes are marked as: * (*p* ≤ 0.05), ** (*p* ≤ 0.005), *** (*p* ≤ 0.0005).

**Figure 6:**
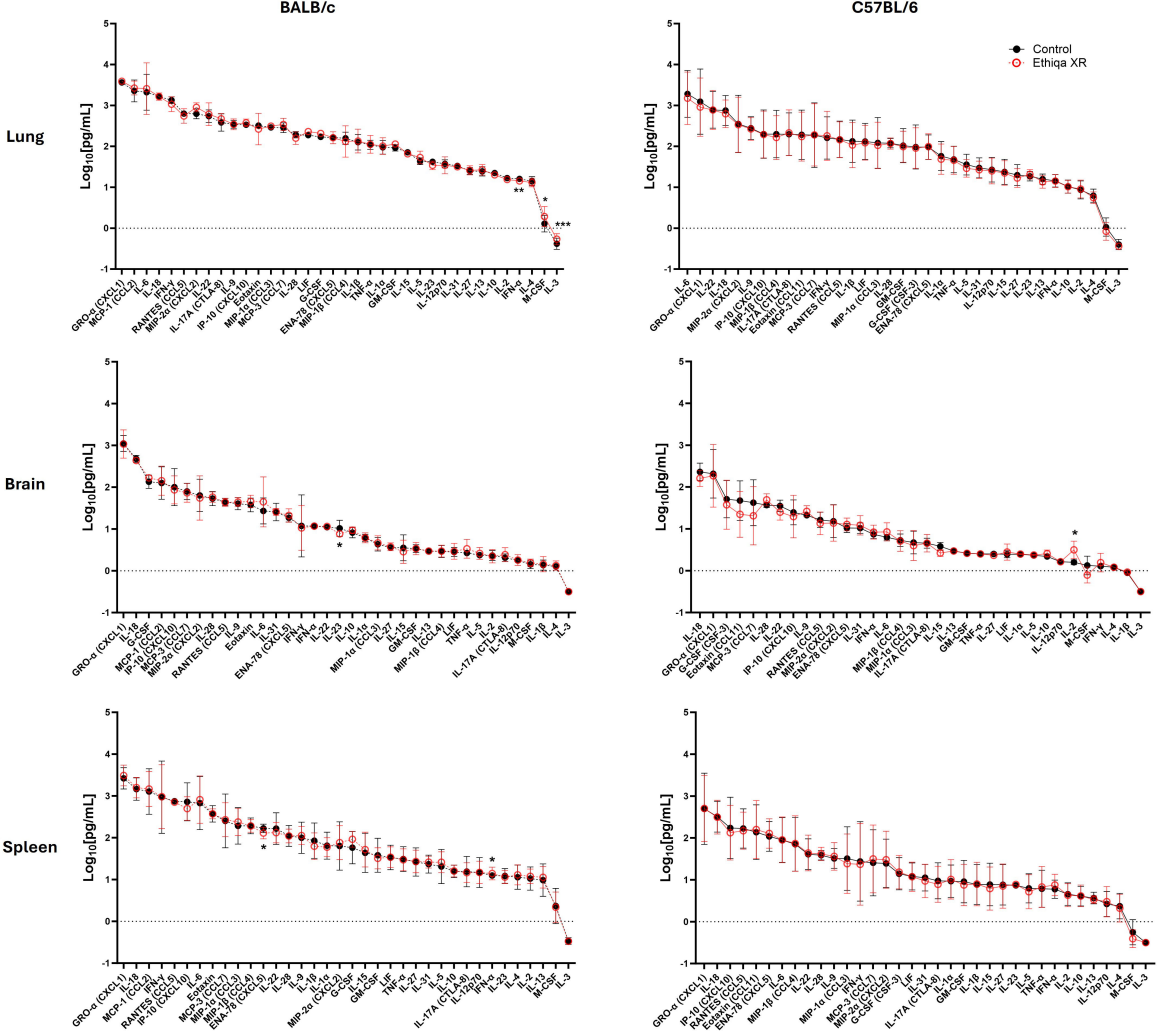
Cytokine response from Ethiqa XR-treated and control BALB/c and C57BL/6 mice post *Y. pestis* challenge. Cytokine multiplex analysis in lung, brain, and spleen homogenates from BALB/c (*n* = 9 per group) and C57BL/6 (*n* = 5 per group) mice 60 h post-challenge. Data (mean ± SD) show log-transformed concentration values. Pairwise treatment groups were compared by linear mixed effects model with no multiplicity adjustment applied. Statistically significant changes are marked as: * (*p* ≤ 0.05), ** (*p* ≤ 0.005), *** (*p* ≤ 0.0005).

To assess the effect of Ethiqa XR on the cytokine response, we employed the same multiplex MagPix analysis previously described to lung, brain, and spleen homogenates (**Figures 5**, **6**). At 24 h, lung, brain, and spleen homogenates from control BALB/c mice challenged with *Y. pestis* CO92 had similar cytokine concentrations than non-challenged control mice (**Figure 2**, BALB/c column; **Figure 5**, 24 h column), suggesting the infection was still in the early stages of development or in the pre-inflammatory stage of the canonical *Y. pestis* mediated biphasic immune response. As expected, by 48 h post-challenge, there was a marked cytokine response compared to the 24 h time point and non-challenged mice, regardless of treatment (**Figures 2A, C, E**; **Figure 5**, 48 h column). Treatment with Ethiqa XR did not markedly change the cytokine responses in lung or brain homogenates at 24 h or 48 h after challenge, with the exception of a few cytokines (**Figure 5**, Lung and Brain rows). In the lung homogenates, at 24 h, MCP-3 (CCL7) and IL-5 were significantly higher in the Ethiqa XR-treated group whereas at 48 h, IL-13, IL-4, and IL-3 were significantly lower (**Figure 5**, Lung row). In brain homogenates from Ethiqa XR-treated mice, we only found a significantly lower concentration of ENA-78 (CXCL5) and MIP-2α (CXCL2) at 24 h and 48 h, respectively, and higher IL-10 at 48 h (**Figure 5**, Brain row). Intriguingly, at 24 h, spleen homogenates from Ethiqa XR-treated mice displayed a trend of lower cytokine response when compared to control: RANTES (CCL5), IL-18, Eotaxin, ENA-78 (CXCL5), MIP-1β (CCL4), MIP-1α (CCL3), and IL-1β whereas LIF and IL-5 were higher (**Figure 5**, 24 h column, Spleen). At 48 h, we found a similar trend: IL-28, IL-9, IL-22, LIF, IL-27, IL-23, IL-4, GM-CSF, IL-2, IL-13, and G-CSF were significantly lower in the Ethiqa-treated group (**Figure 5**, 48 h column, Spleen). Except for IL-23, these differences between groups in the brain were resolved at 60 h post-challenge (**Figure 6**, BALB/c column). Overall, the cytokine response did not markedly change at 60 h compared to 48 h, suggesting the response already peaked by 48 h. In contrast, no significant differences in the cytokine response were detected in lung and spleen homogenates from C57BL/6 mice treated with Ethiqa XR, relative to control 60 h post-challenge. Only IL-2 concentration was higher in brain homogenates from Ethiqa-treated C57BL/6 mice (**Figure 6**, C57BL/6 column, Brain row). These data generated in BALB/c mice suggest that Ethiqa XR may alter the spleen inflammatory response at early stages after *Y. pestis* CO92 challenge.

**Figure 7:**
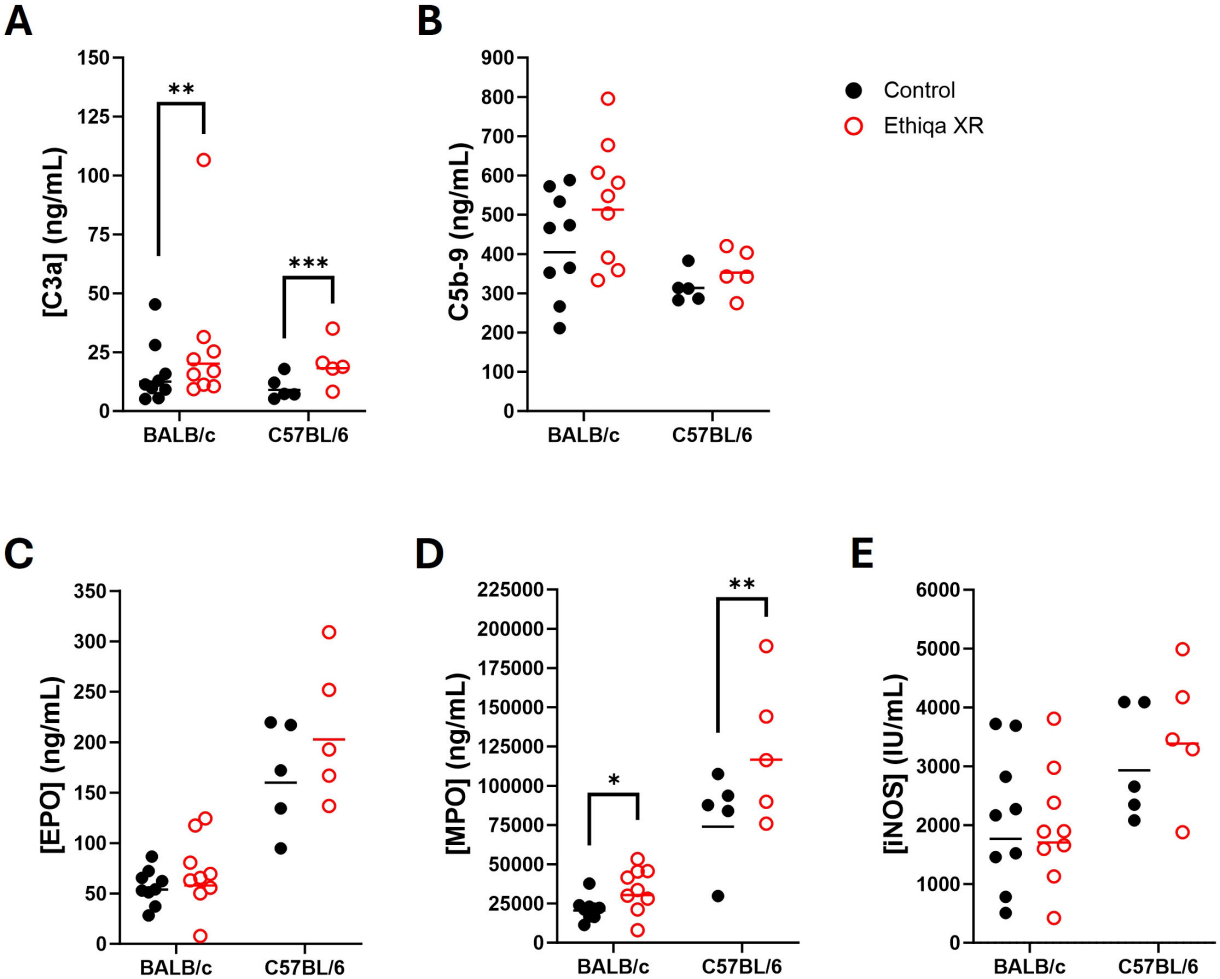
Increased complement and neutrophil activity after Ethiqa XR treatment in *Y. pestis* challenged mice. Serum complement activation measured as the concentration of **(A)** C3a and **(B)** C5b-9 from Ethiqa XR-treated and control BALB/c (*n* = 9 per group) and C57BL/6 (*n* = 5 per group) mice 60 h post-challenge. Horizontal bars show the geomean. Activity of eosinophils, macrophages, and neutrophils measured as the concentration of **(C)** eosinophil peroxidase (EPO), **(D)** myeloxidase (MPO), and **(E)** inducible nitric oxide synthase (iNOS) in lung homogenates from BALB/c (*n* = 9 per group) and C57BL/6 (*n* = 5 per group) mice. Horizontal bars show the geometric mean. Pairwise treatment groups were compared by linear mixed effects model with no multiplicity adjustment applied. Statistically significant changes are marked as: * (*p* ≤ 0.05), ** (*p* ≤ 0.005), *** (*p* ≤ 0.0005).

Next, we measured the concentration of C3a and C5b-9 in the serum to assess complement activation (**Supplementary Figures 2A, B**; **Figures 7A, B**). Since we did not see any significant changes in the cytokine response in the spleens of challenged mice and only a few changes in the lungs (**Figure 6**), we concentrated our EPO, MPO and iNOS analysis in the lung to evaluate the lung tissue as the portal of entry for the bacteria (**Supplementary Figures 2C–E**; **Figures 7C–E**). At 24 h, we did not find any differences between groups in the serum or lung homogenates from BALB/c mice (**Supplementary Figures 2A–E**). At 60 h post-challenge, both BALB/c and C57BL/6 mice treated with Ethiqa XR had significantly higher levels of C3a in the serum compared to control (**Figure 7A**). Although not significant, we also found higher C5b-9 concentration in both strains of Ethiqa XR-treated mice (**Figure 7B**). As expected, we noted an overall higher concentration of C3a and C5b-9 compared to non-challenged mice (**Figures 7A, B** vs **Figures 2A, B**). Interestingly, the concentrations of C3a and C5b-9 in BALB/c mice 24 h post-challenge with *Y. pestis* CO92 were very similar to those from non-challenged mice (**Supplementary Figures 2A, B** vs **Figures 3A, B**). No significant changes were detected in EPO concentrations in lung homogenates treated with Ethiqa XR relative to control, although C57BL/6 mice treated with Ethiqa XR had slightly higher concentrations (**Figure 7C**). Both mouse strains showed significant higher concentrations of MPO in lung homogenates when treated with Ethiqa XR (**Figure 7D**), suggesting an increase in neutrophil activity. In contrast, no significant differences were found in macrophage activity (iNOS concentration) between experimental groups from either BALB/c or C57BL/6 lung homogenates (**Figure 7E**). These results suggest that Ethiqa XR may affect complement and neutrophil activity in the sera and lungs of BALB/c and C57BL/6 mice challenged with *Y. pestis*.

### 3.3 BALB/c and more markedly C57BL/6 mice challenged with aerosolized *B. pseudomallei* show an increased pro-inflammatory response when treated with Ethiqa XR

Next, we investigated the effects of treatment with Ethiqa XR in mice challenged with *B. pseudomallei* K96243 following the experimental design described in **Figure 1C**.

**Figure 8:**
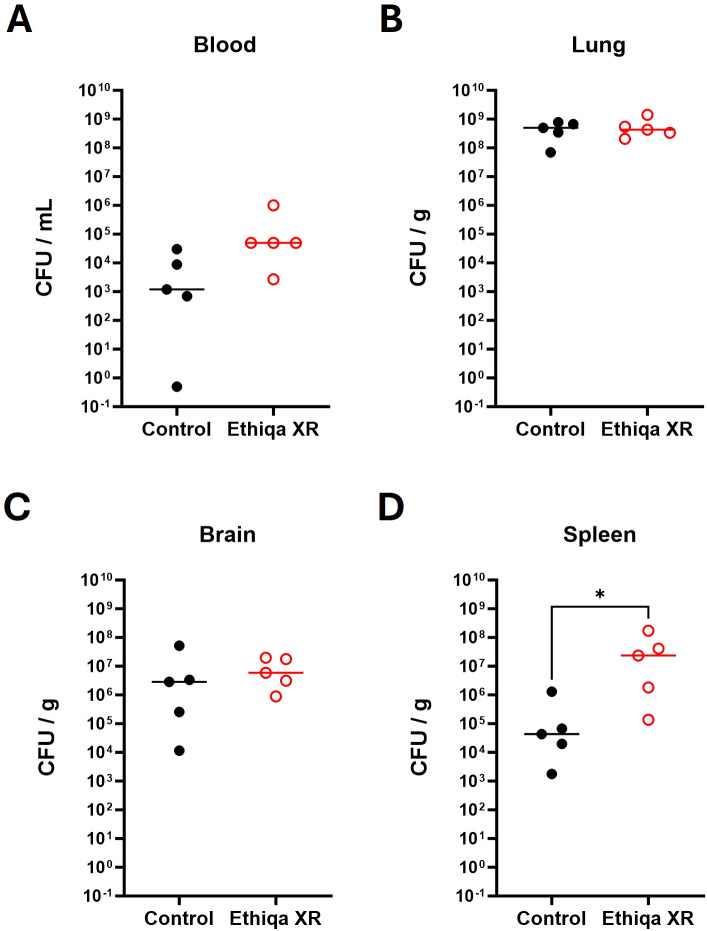
Increase of splenic bacterial burden after Ethiqa XR treatment in C57BL/6 mice post *B. pseudomallei* challenged mice. The bacterial burden of C57BL/6 mice in **(A)** blood **(B)** lung, **(C)** brain, and **(D)** spleen homogenates (*n* = 5 per group) 72 h post challenge with *B. pseudomallei* K96243. Horizontal line shows the median. Statistically significant changes from unpaired nonparametric Mann-Whitney tests are marked as: * (*p* ≤ 0.05).

The bacterial burden in the blood of C57BL/6 mice, as measured by CFU/mL, was appreciably higher in mice treated with Ethiqa XR (100% bacteremic) compared to control group (80% bacteremic) (**Figure 8A**). We did not find any significant difference in the bacterial burden (CFU/g) in the lung or brain from mice treated with Ethiqa XR compared to control (**Figures 8B, C**). But, relative to the control, there was a significantly higher CFU/g burden in the spleen from the Ethiqa XR-treated group (**Figure 8D**).

**Figure 9:**
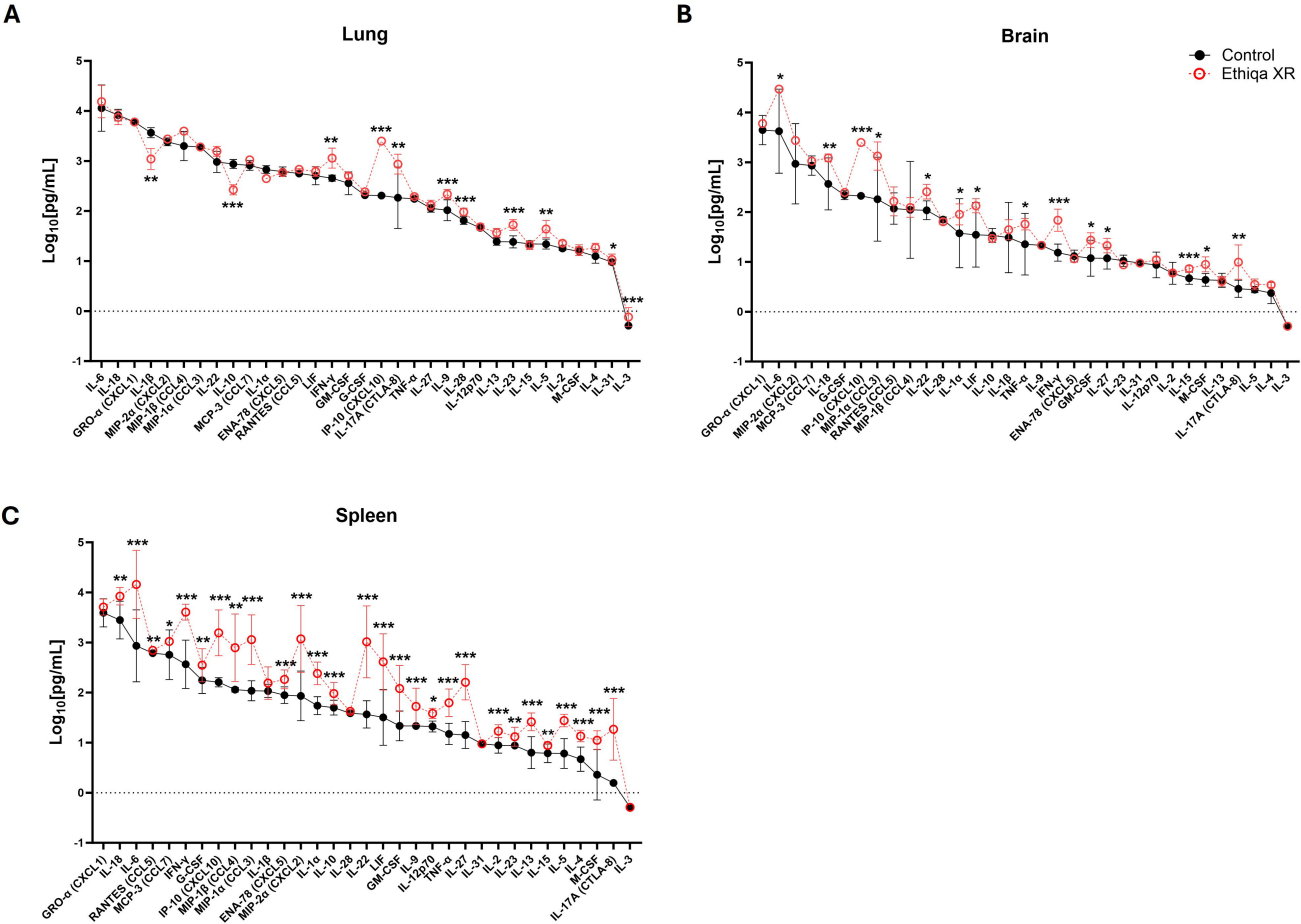
Cytokine response disruption following treatment with Ethiqa XR in C57BL/6 mice post *B. pseudomallei* challenge. Cytokine response in **(A)** lung, **(B)** brain, and **(C)** spleen homogenates (*n* = 5 per group) 72 h post *B. pseudomallei* K96243 challenge in Ethiqa XR-treated and untreated C57BL/6 mice. Data (mean ± SD) show log-transformed concentration values. Pairwise treatment groups were compared by linear mixed effects model with no multiplicity adjustment applied. Statistically significant changes are marked as: * (*p* ≤ 0.05), ** (*p* ≤ 0.005), *** (*p* ≤ 0.0005).

Multiplex MagPix analysis of lung, brain, and spleen homogenates from Ethiqa XR-treated group revealed an overall increased pro-inflammatory cytokine response compared to control (**Figure 9**). The concentrations of IL-1β and IL-10 from lung homogenates were significantly lower in the Ethiqa XR group while IFN-γ, IP-10 (CXCL10), IL-17A (CTLA-8), IL-9, IL-28, IL-23, IL-5, IL-31, and IL-3 were higher, relative to control (**Figure 9A**). In the brain, relative to control, the Ethiqa XR-treated group had a significantly higher concentration of the following cytokines: IL-6, IL-18, IP-10 (CXCL10), MIP-1α (CCL3), IL-22, IL-1α, LIF, TNF-α, IFN-γ, GM-CSF, IL-27, IL-15, M-CSF, and IL-17A (CTLA-8) (**Figure 9B**).

Likewise, the cytokine response in the spleen was markedly higher in the Ethiqa XR group compared to control (**Figure 9C**). More than 80% (28/33) of the cytokines were significantly higher in spleens of mice treated with Ethiqa XR, relative to control (**Figure 9C**). These results align with higher bacterial burden in the spleens and also the increased cytokine response from lung and spleen homogenates of non-challenged C57BL/6 mice treated with Ethiqa XR described above (**Figure 2**, C57BL/6 panel).

**Figure 10:**
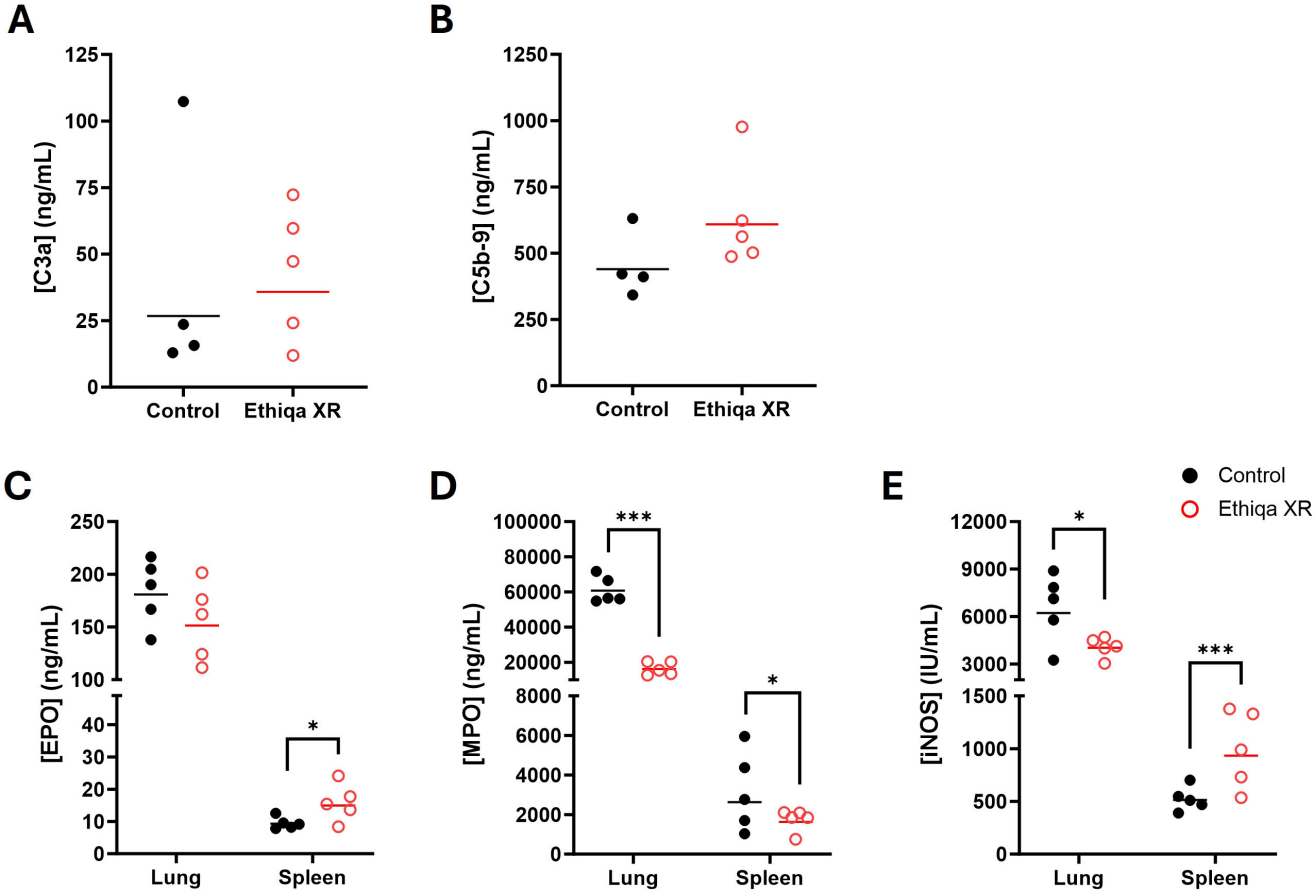
Alteration in eosinophil, macrophage, and neutrophil activity following Ethiqa XR treatment in C57BL/6 mice post *B. pseudomallei* challenge. Complement activation measured as the serum concentration of **(A)** C3a and **(B)** C5b-9 from Ethiqa XR-treated (*n* = 4) and control (*n* = 5) C57BL/6 mice 72 h post *B. pseudomallei* K96243 challenge. Eosinophil, macrophage, and neutrophil activity measured as the concentration of **(C)** eosinophil peroxidase (EPO), **(D)** myeloxidase (MPO), and **(E)** inducible nitric oxide synthase (iNOS) from the specified tissue homogenates (*n* = 5 per group). Horizontal bars show the geometric mean. Pairwise treatment groups were compared by linear mixed effects model. Statistically significant changes are marked as: * (*p* ≤ 0.05), *** (*p* ≤ 0.0005).

To further investigate this pro-inflammatory response from Ethiqa XR-treated mice, we measured C3a and C5b-9 concentration in the serum (**Figures 10A, B**). Surprisingly, C3a concentration was similar between experimental groups (**Figure 10A**). The concentration of the terminal complex C5b-9 was higher in the Ethiqa XR-treated group, but not statistically significant (marginal *p* value = 0.0863) (**Figure 10B**). As previously described, we also measured the concentration of EPO, MPO, and iNOS in lung and spleen homogenates (**Figures 10C–E**). Despite the highly pro-inflammatory cytokine response in the lung of Ethiqa XR-treated C57BL/6 mice described above (**Figure 9**), we found an overall lower concentration of EPO, MPO, and iNOS in the lungs (**Figures 10C–E**, Lung). Consistent with the even more pro-inflammatory spleen cytokine response, we found a higher concentration of EPO and iNOS in the spleen homogenates from Ethiqa XR-treated mice, but MPO was significantly lower in Ethiqa XR-treated mice (**Figures 10C–E**, Spleen). These results suggest that in C57BL/6 mice, during inhalational melioidosis, Ethiqa XR may have a net suppressive effect on macrophage activity in the lung and a dampening effect on neutrophil recruitment despite the pro-inflammatory signals that normally link high bacterial burden to high neutrophil content in tissues.

Next, we investigated whether the Ethiqa XR effects observed in C57BL/6 mice are also observed in BALB/c mice challenged with aerosolized *B. pseudomallei* K96243 (**Figure 1C**). It has been previously reported that BALB/c mice develop a more acute infection with *B. pseudomallei* compared to C57BL/6 mice (12–15). Therefore, we harvested blood, lungs, spleen, and brain of BALB/c mice at 60 h and 72 h post-challenge (**Figure 1C**).

**Figure 11:**
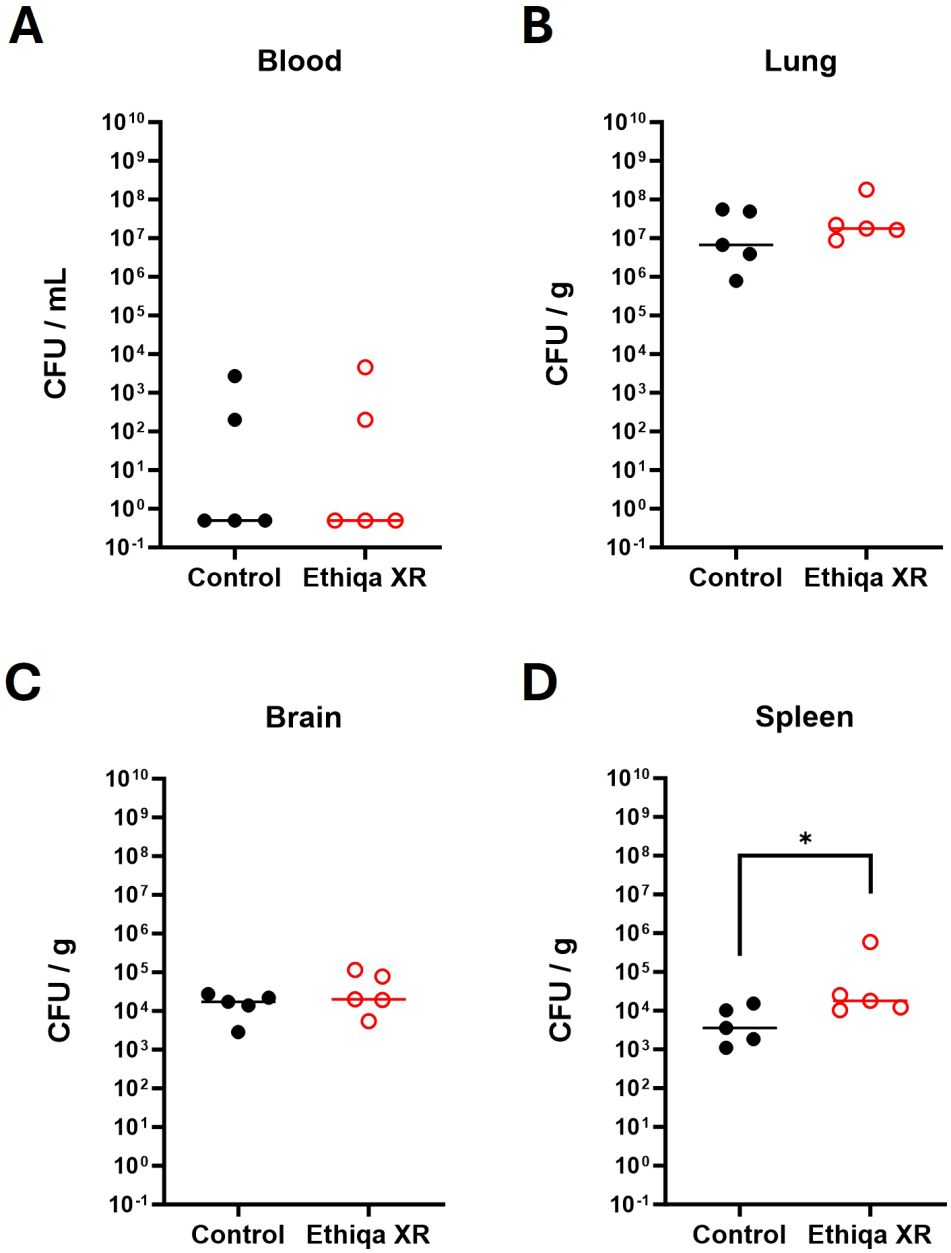
Increased bacterial burden of Ethiqa XR-treated BALB/c mice post *B. pseudomallei* challenge. Bacterial burden of BALB/c mice in **(A)** blood **(B)** lung, **(C)** brain, and **(D)** spleen homogenates (*n* = 5 per group) mice 60 h post-challenge with *B. pseudomallei* K96243. Horizontal line shows the median. Statistically significant changes from unpaired nonparametric Mann-Whitney tests are marked as: * (*p* ≤ 0.05).

At 60 h, the bacterial burden in the blood (CFU/mL) for both Ethiqa XR-treated and control groups were remarkably similar. Only 40% (2/5) of mice were bacteremic in each group (**Figure 11A**). The same results were observed at 72 h, where 75% (3/4) of mice were bacteremic regardless of Ethiqa XR treatment (**Supplementary Figure 3A**). The bacterial burdens in lung and brain homogenates were similar in both groups and timepoints (**Figures 11B, C**; **Supplementary Figures 3B, C**). The bacterial burden in spleen homogenates was significantly higher in the Ethiqa XR-treated mice compared to control BALB/c mice at 60 h (**Figure 11D**). However, this difference was not observed at 72 h (**Supplementary Figure 3D**).

**Figure 12:**
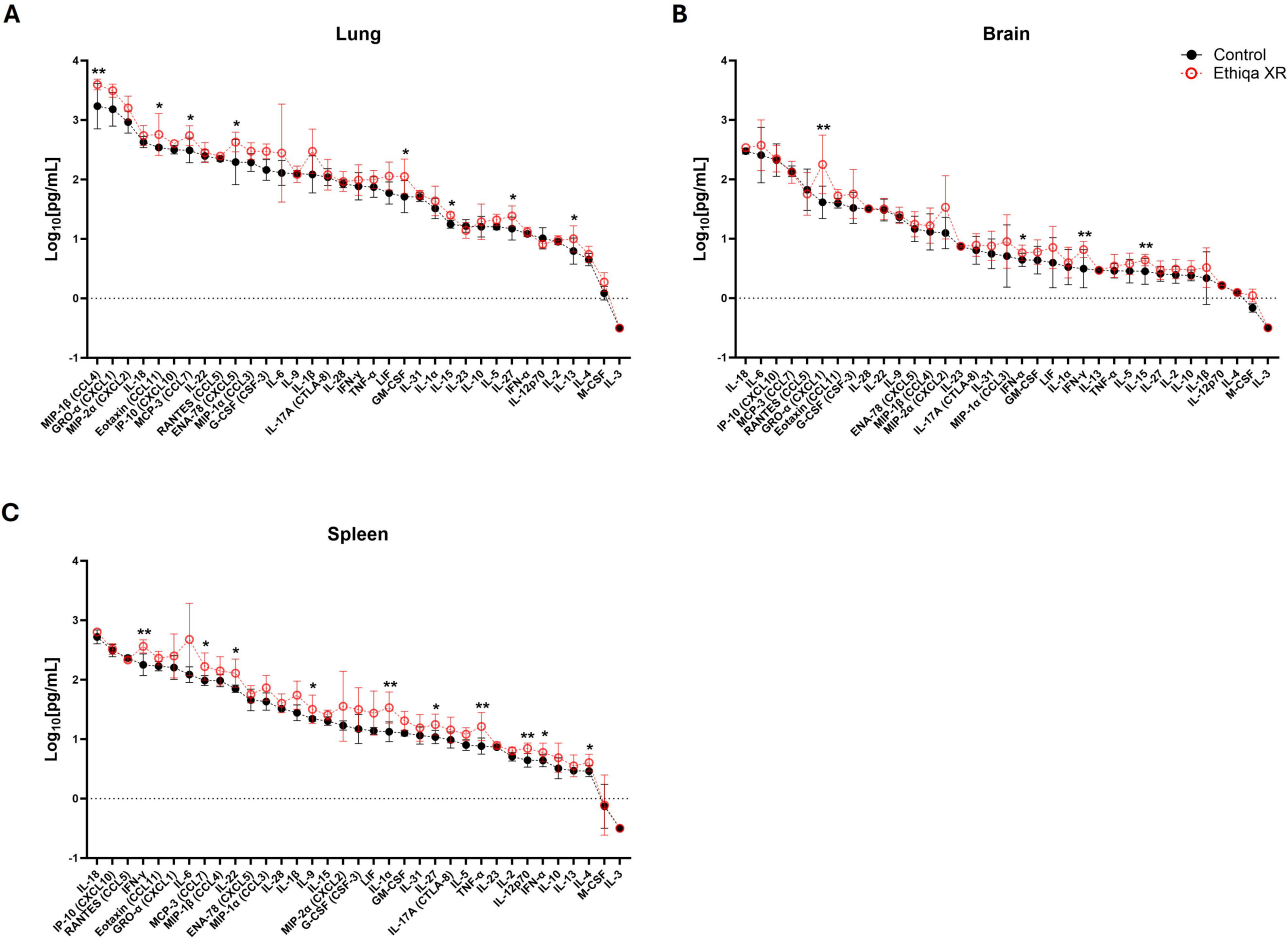
Alteration in the cytokine response in Ethiqa XR- treated BALB/c mice post *B. pseudomallei* challenge. Cytokine response in **(A)** lung, **(B)** brain, and **(C)** spleen homogenates in Ethiqa XR treated and control BALB/c mice (*n* = 5 per group) 60 h after *B. pseudomallei* K96243 challenge. Data (mean ± SD) show log-transformed concentration values. Pairwise treatment groups were compared by linear mixed effects model with no multiplicity adjustment applied. Statistically significant changes are marked as: * (*p* ≤ 0.05), ** (*p* ≤ 0.005).

MagPix multiplex analysis was employed to assess the cytokine response in lung, brain and spleen homogenates. Relative to C57BL/6 mice, BALB/c mice treated with Ethiqa XR did not show a robust cytokine response when compared with control (**Figure 12**, **Supplementary Figure 4**). At 60 h post-challenge, the lung homogenates from the Ethiqa XR-treated group had a significantly higher concentration of MIP-1β (CCL4), Eotaxin (CCL11), MCP-3 (CCL7), ENA-78 (CXCL5), GM-CSF, IL-15, IL-27, and IL-13, compared to control (**Figure 12A**). In the brain, only GRO-α (CXCL1), IFN-α, IFN-γ, and IL-15 were significantly higher in the Ethiqa XR group (**Figure 12B**). Although not as marked as C57BL/6 mice, we did find an overall higher concentration of pro-inflammatory cytokines in the spleen of Ethiqa XR-treated BALB/c mice: IFN-γ, MCP-3 (CCL7), IL-22, IL-9, IL-1α, IL-27, TNF-α, IL-12p70, IFN-α, and IL-4 (**Figure 12C**). This is consistent with a higher bacterial burden in the spleen at 60 h described above (**Figure 11D**). However, most of these differences were resolved by 72 h post-infection (**Supplementary Figure 4**). These results align with the pro-inflammatory response in mice treated with Ethiqa XR and challenged with *B. pseudomallei*.

**Figure 13:**
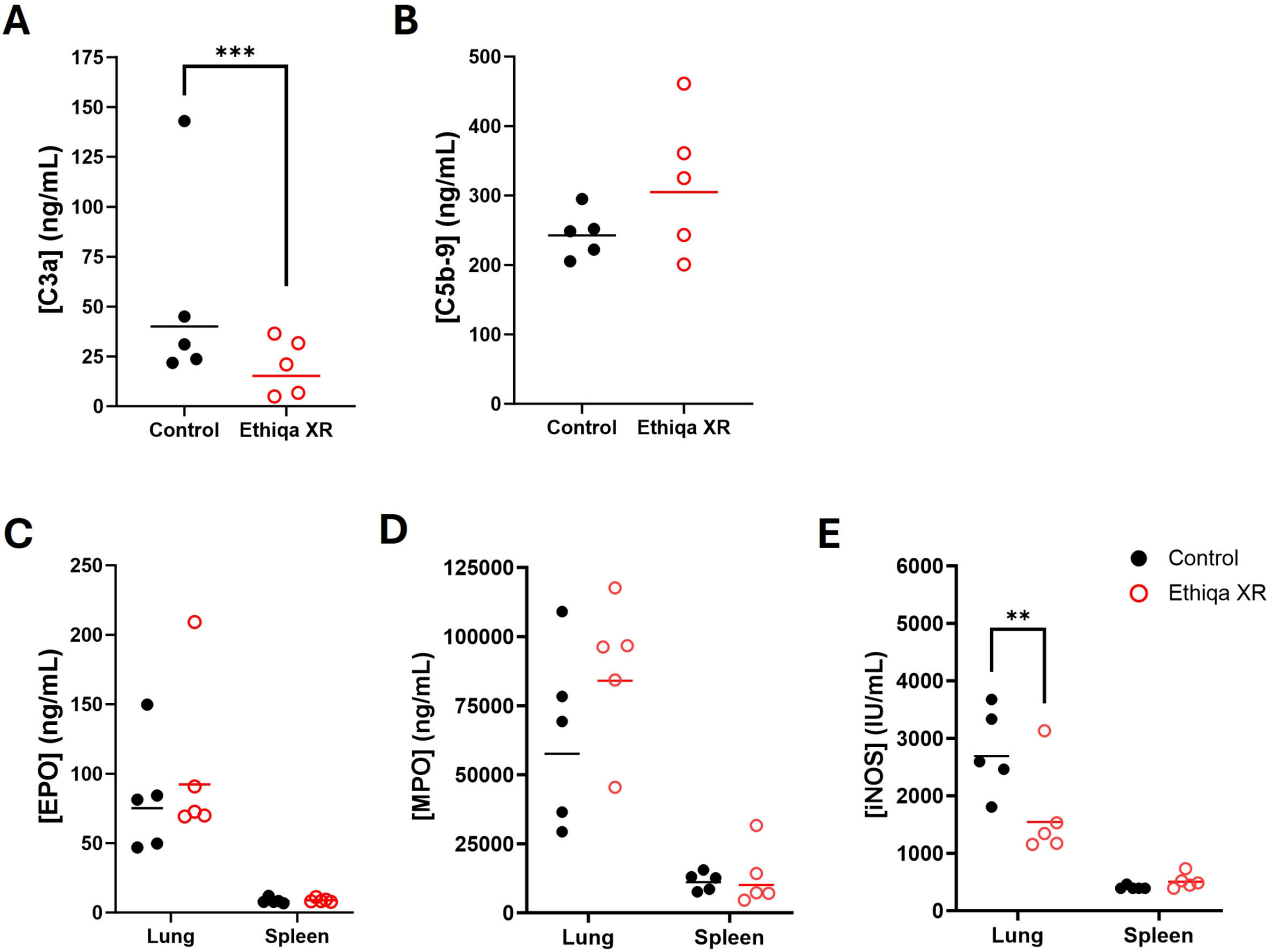
Disruption in complement activation and macrophage activity after Ethiqa XR- treatment in BALB/c mice post *B. pseudomallei* challenge. Serum complement activation measured as the concentration of **(A)** C3a and **(B)** C5b-9 in Ethiqa XR- treated and control BALB/c mice (*n* = 5 per group) 60 h post *B. pseudomallei K96243* challenge. Eosinophil, macrophage, and neutrophil activity as measured by the concentration of **(C)** eosinophil peroxidase (EPO), **(D)** myeloxidase (MPO), and **(E)** inducible nitric oxide synthase (iNOS) from the specified tissue homogenates. Horizontal bars show the geometric mean. Pairwise treatment groups were compared by linear mixed effects model. Statistically significant changes are marked as: ** (*p* ≤ 0.005), *** (*p* ≤ 0.0005).

To investigate complement activation, we measured the C3a and C5b-9 levels in the serum (**Figures 13A, B**; **Supplementary Figures 5A, B**). The concentration of C3a was lower in Ethiqa XR-treated mice both at 60 h and 72 h (**Figure 13A**m **Supplementary Figure 5A**). However, although not significant, the concentration of C5b-9 was higher in Ethiqa XR-treated mice at both time points (**Figure 13B**, **Supplementary Figure 5B**). We also measured the concentration of EPO, MPO, and iNOS in lung and spleen homogenates (**Figures 13C–E**; **Supplementary Figures 5C–E**). We did not find significant differences in the concentration of EPO in the lungs or the spleen from any of the experimental groups (**Figure 13C**; **Supplementary Figure 5C**). The concentration of MPO in the lung of Ethiqa XR-treated mice was higher at 60 h (**Figure 13B**) but lower at 72 h (**Supplementary Figure 5B**). However, these differences were not statically significant (marginal *p* value = 0.0770). Similarly, we did not find any significant change in the MPO concentration in the spleen from both experimental groups (**Figure 13B**, **Supplementary Figure 5B**). Consistent with C57BL/6, we found that BALB/c mice treated with Ethiqa XR also have a significantly lower concentration of iNOS in lungs at both 60 h and 72 h (**Figure 13E**; **Supplementary Figure 5E**, Lung panels). No significant differences were found in the concentration of iNOS in the spleen (**Figure 13E**; **Supplementary Figure 5E**, Spleen panels). Overall, these results suggest that the effects of Ethiqa XR are influenced by both the mouse strain and the bacterium.

## 4 Discussion

The experiments described here were designed to assess the impact of the extended-release formulation of buprenorphine (Ethiqa XR) on acute inhalational infections with biothreat agents and our goal was to determine to what extent the analgesic impact would have on downstream data interpretations. Not surprisingly, the two mouse strains yielded different results.

Despite BALB/c mice showing a higher, but still overall low, inflammatory response in the brain compared to C57BL/6 (**Figures 2A, B**), our data show that C57BL/6 mice have an overall stronger pro-inflammatory response in the lung and spleen compared to BALB/c when treated with Ethiqa XR (**Figures 2C–F**). Interestingly, the inflammatory response from C57BL/6 was more robust in the lungs than in the spleen. C5b-9 (membrane attack complex) has been implicated in the activation of the inflammasome through sublytic signaling pathways (16, 17). Consistently, the concentration of the complement terminal complex C5b-9 in the serum was also higher in C57BL/6 mice receiving Ethiqa XR treatment (**Figure 3B**). It is known that opioid-receptor mediated analgesia can induce respiratory depression in mice (18, 19). Although buprenorphine has milder respiratory depression effects than other opioids (11), these data reported here suggest that C57BL/6 mice are more susceptible to this analgesic.

Treatment with Ethiqa XR has a suppressive effect in lung macrophage activity (**Figures 3E**, **10E**, **13E**; **Supplementary Figure 5E**). Once again, C57BL/6 appears to be more susceptible than BALB/c. Other studies have shown that buprenorphine reduces the monocytes CCL2-mediated chemotactic response (20). Furthermore, a more recent study showed that 8-week old male C57BL/6J mice treated daily for 72 h with buprenorphine prior to infection with EcoHIV (a chimeric HIV that replicates in mice) (21, 22) were protected from developing neurocognitive impairment possibly due to fewer brain inflammatory (Ly6C^hi^) monocytes (23). Buprenorphine reduced the expression of IL-6, TNF-a, and IL-12 in M1-polarized macrophages derived from either human umbilical cord or murine peripheral blood (24). In contrast, buprenorphine increased the expression of Ym1 and Fizz1 of M2-polarized macrophages (24), suggesting an inhibitory role in inflammation. However, we also found a significant increase in markers for macrophage and as eosinophil, activity in the spleen (**Figures 10C, E**). We attribute these responses to the high bacterial burden in the Ethiqa XR-treated C57BL/6 mice after *B. pseudomallei* challenge (**Figure 8D**). This apparent dual effect in the macrophage activity suggests that Ethiqa XR modulatory impact may vary based on organ, treatment time, and pathogen.

However, these suppressive effects are not observed in either mouse strain when challenged with *Y. pestis* CO92 (**Figure 7E**). Although *Y. pestis* can inhibit the neutrophil production of antimicrobial extracellular vesicles (25), the neutrophil activity in the lung was elevated in both mouse strains challenged with *Y. pestis* upon treatment with Ethiqa XR (**Figure 7D**). It is worth noting that a marker for neutrophil activity in the lungs was lower in non-challenged BALB/c mice and *B. pseudomallei* challenged C57BL/6 mice treated with Ethiqa XR (**Figures 3D**, **10D**). Moreover, there was a more prominent elevation of C3a and C5b-9 in the serum of both strains of mice after Ethiqa XR treatment and *Y. pestis* challenge (**Figure 7A**). This suggests that Ethiqa XR may have differential effects based upon the bacterium used for the infection. For example, we found that treatment with Ethiqa XR noticeably increased the clinical signs (e.g. labored breath, ruffled fur, and abnormal behavior such as less peer interaction) of mice challenged with *B. pseudomallei* (data not shown), promoted higher bacterial dissemination to the spleen and induced high pro-inflammatory responses in the same organ (**Figures 8D**, **9**, **11D**) for both mouse strains. Despite similar bacterial burden in the lungs and brains from both groups (**Figures 8B, C**, **11B, C**), there was a higher inflammatory response in these organs upon treatment with Ethiqa XR. These results resemble the higher pro-inflammatory response of Ethiqa XR-treated mice in the absence of challenge (**Figure 2**). Therefore, these responses are likely attributable to Ethiqa XR treatment.

Data presented in this current report also suggest Ethiqa XR may have different impacts on the immune response based upon the animal used in the model. As discussed earlier, non-challenged C57BL/6 mice appear to have a higher pro-inflammatory response upon treatment with Ethiqa XR, relative to BALB/c (**Figures 2C, F**). These differences become more apparent in the context of *B. pseudomallei* challenge. While both strains of mice showed a higher bacterial burden in the spleen when treated with Ethiqa XR (**Figures 8D**, **11D**), the differences in cytokine response in C57BL/6 at 72 h were substantially higher compared to BALB/c at 60 h (**Figure 9** vs **Figure 12**). The differences between control and Ethiqa XR-treated BALB/c mice are not observed at 72 h (**Supplementary Figure 4**). In addition, C57BL/6 mice had evidence of impaired neutrophil activation and/or recruitment at sites of *B. pseudomallei* infection after Ethiqa XR treatment, based on lower MPO levels in lungs and spleen (**Figure 10**), which was not seen in BALB/c mice (**Figure 13**) or after *Y. pestis* challenge (**Figure 7**). These findings further support previous studies demonstrating differences between these two mouse strains in the context of melioidosis (12–15).

We were, however, somewhat surprised that the impacts of analgesia appeared to be different using two gram-negative bacteria in acute inhalational models. The differences in bacterial pathogenesis between *Y. pestis* and *B. pseudomallei* are numerous, but aerosol exposure to either bacterium generally results in naïve animals succumbing to disease or meeting early endpoint euthanasia criteria by day 4 post-exposure. However, even during this short infection time, the distinct bacterial pathogenesis of each bacterium may have contributed to different outcomes after treatment with Ethiqa XR. For example, the understood immunomodulation associated with *Y. pestis* (e.g., Yop effector proteins) may have resulted in a less apparent immune profile differential between control mice and Ethiqa XR-treated mice (26–30). Additionally, the number of bacteria used in these experiments can be quite different as well. For example, the LD_50_ for *Y. pestis* CO92 in BALB/c mice is approximately 6.8x10^4^ CFU and in C57BL/6 the LD_50_ is approximately 2.9x10^4^ (10) (data not shown). Whereas for *B. pseudomallei* K96243, the LD_50_ is approximately 25 CFU for BALB/c mice and 360 CFU for C57BL/6 mice (9). There is also additional inherent variability because these bacteria were delivered via small-particle aerosolization techniques, and thus the number of LD_50_ equivalents delivered may also lead to some of the differences or lack of differences we describe here. However, it is important to note that despite these potential differences that may be attributed to dosing or documented disparities associated with disease and bacterial pathogenesis between pneumonic plague and inhalational melioidosis, the disease severity at approximately 60–72 hours post-exposure to aerosolized bacteria can be remarkably similar in naïve control animals, independent of mouse strain.

These studies underscore the importance of examining even subtle differences associated with analgesic administration in animal models of infectious diseases. However, we acknowledge several limitations associated with our experimental designs. Initially, we chose to only to use female mice, as these are our traditional animal subjects. As we have previously documented, there are sex-related differences associated with our disease models (12, 31), but for this proof-of-concept study we focused on female mice as they are most pertinent to our work and provide the necessary data to investigate the impacts of analgesic intervention. Other sex-related concerns include aggression, hormone differences, and self-mutilation observed in males associated with disease manifestations of melioidosis that are not observed in females. We also did not examine the proprietary vehicle formulation used in the time-release formulation on its own, nor did we analyze the immunological impacts of the injection procedure in the absence of analgesic being administered. Our objective was to generate data dealing with immunological dysregulation associated with administering this specific analgesic via subcutaneous injection, and thus we viewed all of these aspects as components of analgesia administration (32). Lastly, the extensive parallel tests across cytokines, tissues, time points, mouse strains, etc. raises a multiplicity concern and may increase the risk of false positive findings. As these analyses were exploratory and hypothesis-generating, no formal multiplicity adjustment was applied. The consistency of certain biological trends across related endpoints provides supportive evidence; however, these results require further validation in other *in vivo* disease models. Therefore, we are not reporting mechanistic or causation data as the purpose of these studies was to demonstrate differences in certain aspects of the immunological response that may be altered following intervention with analgesics (i.e., Ethiqa XR) that potentially mask or obscure the intended results during various studies. As the use of laboratory animal models continues to evolve, it is imperative to understand these differences before implementing analgesic guidelines or recommendations, even for control animals in order to avoid downstream misinterpretation of data. All of these differences in immunological response to bacteria and/or altered bacterial pathogenesis induced by analgesia administration could impact future medical countermeasure development against threats important to both the biodefense and public health research.

## Data Availability

The original contributions presented in the study are included in the article/**Supplementary Material**. Further inquiries can be directed to the corresponding authors.

## Author disclaimer

The contributions of CR, MD, MH, TW, WZ, AS, YH-V, and EM, affiliated with Team Chenega Professional & Technical Service; JQ, affiliated with Laulima Government Solutions, LLC; and CX, affiliated with Oak Ridge Institute for Science and Education, were made in the capacity of U.S. Government contractors. This does not constitute an endorsement by the U.S. Government of this or any other contractor. The opinions, interpretations, conclusions, and recommendations presented are those of the author and are not necessarily endorsed by the U.S. Army or the Department of Defense.

## Supplementary material

Supplementary Figure 1 Bacterial burden assessment of Ethiqa XR- and control BALB/c mice 24 h and 48 h post-challenge with *Y. pestis* CO92. Scatter plots **(A-D)** show the bacterial burden in **(A)** blood and **(B)** lung, **(C)** brain, and **(D)** spleen homogenates (*n* = 7 per group). Horizontal line shows the median.

Supplementary Figure 2 ELISA analysis of complement, eosinophil, macrophage, and neutrophil activity of control or Ethiqa XR-treated BALB/c mice 24 h post-challenge with *Y. pestis* CO92. Scatter plots show the serum (*n* = 7 per group) concentration of **(A)** C3a and **(B)** C5b-9 as a measure of complement activation. Scatter plots **(C-E)** show the analysis of eosinophil, macrophage, and neutrophil activity by measuring the concentration of **(C)** eosinophil peroxidase (EPO), **(D)** myeloxidase (MPO), and **(E)** inducible nitric oxide synthase (iNOS) from the lung homogenates (*n* = 7 per group). Horizontal bars show the geometric mean.

Supplementary Figure 3 Assessment of bacterial burden of Ethiqa XR-treated and control BALB/c mice 72 h post-challenge with *B. pseudomallei* K96243. Scatter plots (**A–D**) show the bacterial burden of BALB/c mice in **(A)** blood **(B)** lung, **(C)** brain, and **(D)** spleen homogenates (*n* = 5 per group). Scatter plots **(E-H)** show the bacterial burden in **(E)** blood, **(F)** lung, **(G)** brain, and **(H)** spleen homogenates (*n* = 4 per group). Horizontal line shows the median.

Supplementary Figure 4 Luminex analysis of the cytokine response from Ethiqa XR-treated and control BALB/c mice 72 h after *B. pseudomallei* K96243 challenge. Graphs show cytokine multiplex analysis in **(A)** lung, **(B)** brain, and **(C)** spleen homogenates (*n* = 4 per group). Data (mean ± SD) show log-transformed concentration values. Pairwise treatment groups were compared by linear mixed effects model with no multiplicity adjustment applied. Statistically significant changes are marked as: * (*p* ≤ 0.05), ** (*p* ≤ 0.005).

Supplementary Figure 5 ELISA analysis of complement, eosinophil, macrophage, and neutrophil activity of Ethiqa XR-treated and control BALB/c mice 72 h after *B. pseudomallei* K96243 challenge. Scatter plots **(A, B)** show complement activation measured as the serum (*n* = 4 per group) concentration of **(A)** C3a and **(B)** C5b-9. Scatter plots **(C, E)** show the analysis of eosinophil, macrophage, and neutrophil activity by measuring the concentration of **(C)** eosinophil peroxidase (EPO), **(D)** myeloxidase (MPO), and **(E)** inducible nitric oxide synthase (iNOS) from the specified tissue (*n* = 4 per group) homogenates. Horizontal bars show the geometric mean. Pairwise treatment groups were compared by linear mixed effects model. Statistically significant changes are marked as: ** (*p* ≤ 0.005).
