# Supplementary figures and images for: Buprenorphine extended-release (Ethiqa XR) impacts the immunological response in mice exposed to aerosolized Burkholderia pseudomallei or Yersinia pestis
Source: Front Immunol. 2026 May 13;17:1823747. doi: 10.3389/fimmu.2026.1823747 (PMC13212136; doi:10.3389/fimmu.2026.1823747)

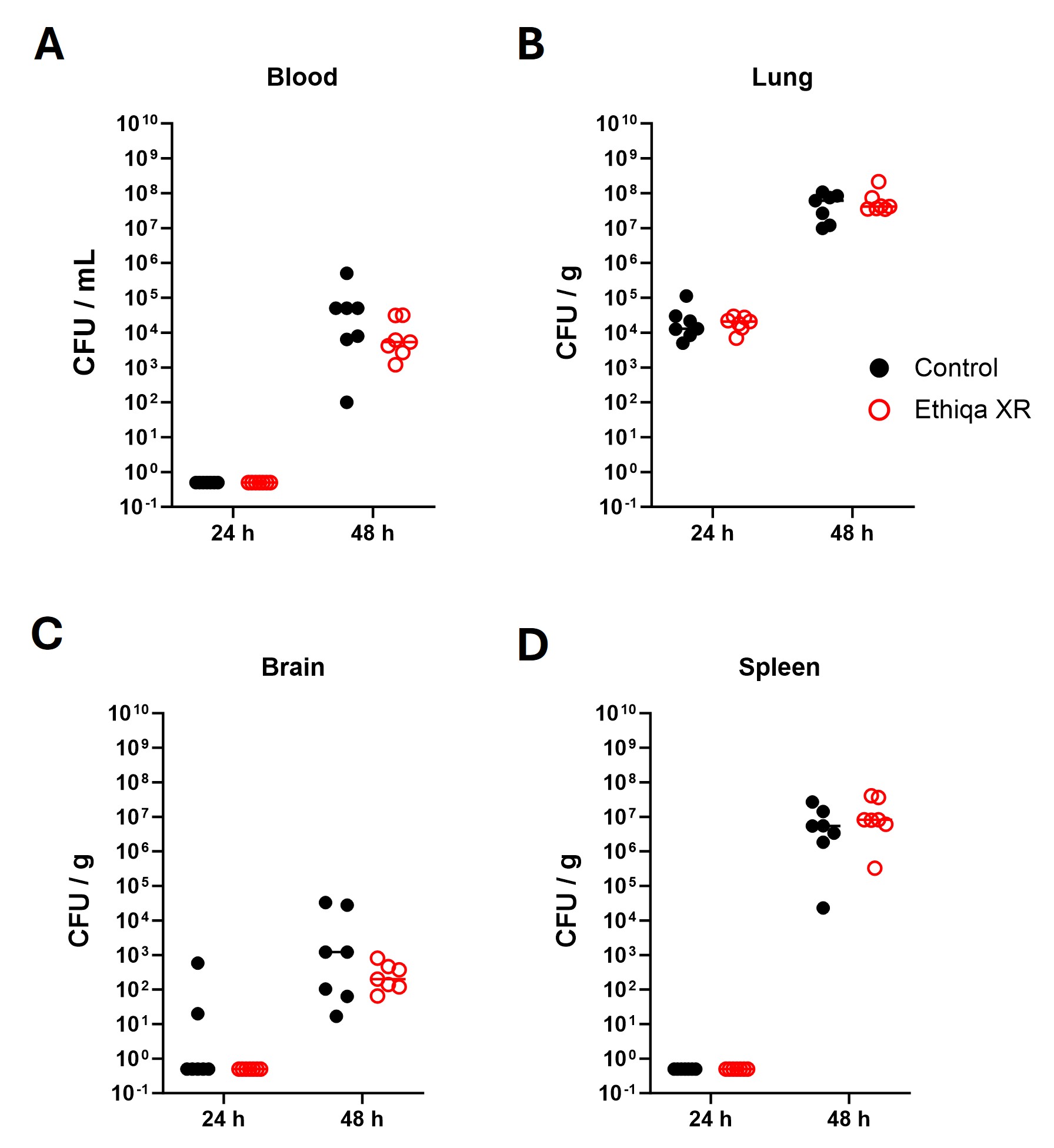

Supplement: Supplementary Figure 1 — Bacterial burden assessment of Ethiqa XR- and control BALB/c mice 24 h and 48 h post-challenge with Y. pestis CO92. Scatter plots (A-D) show the bacterial burden in (A) blood and (B) lung, (C) brain, and (D) spleen homogenates (n = 7 per group). Horizontal line shows the median. [file Image1.jpeg]

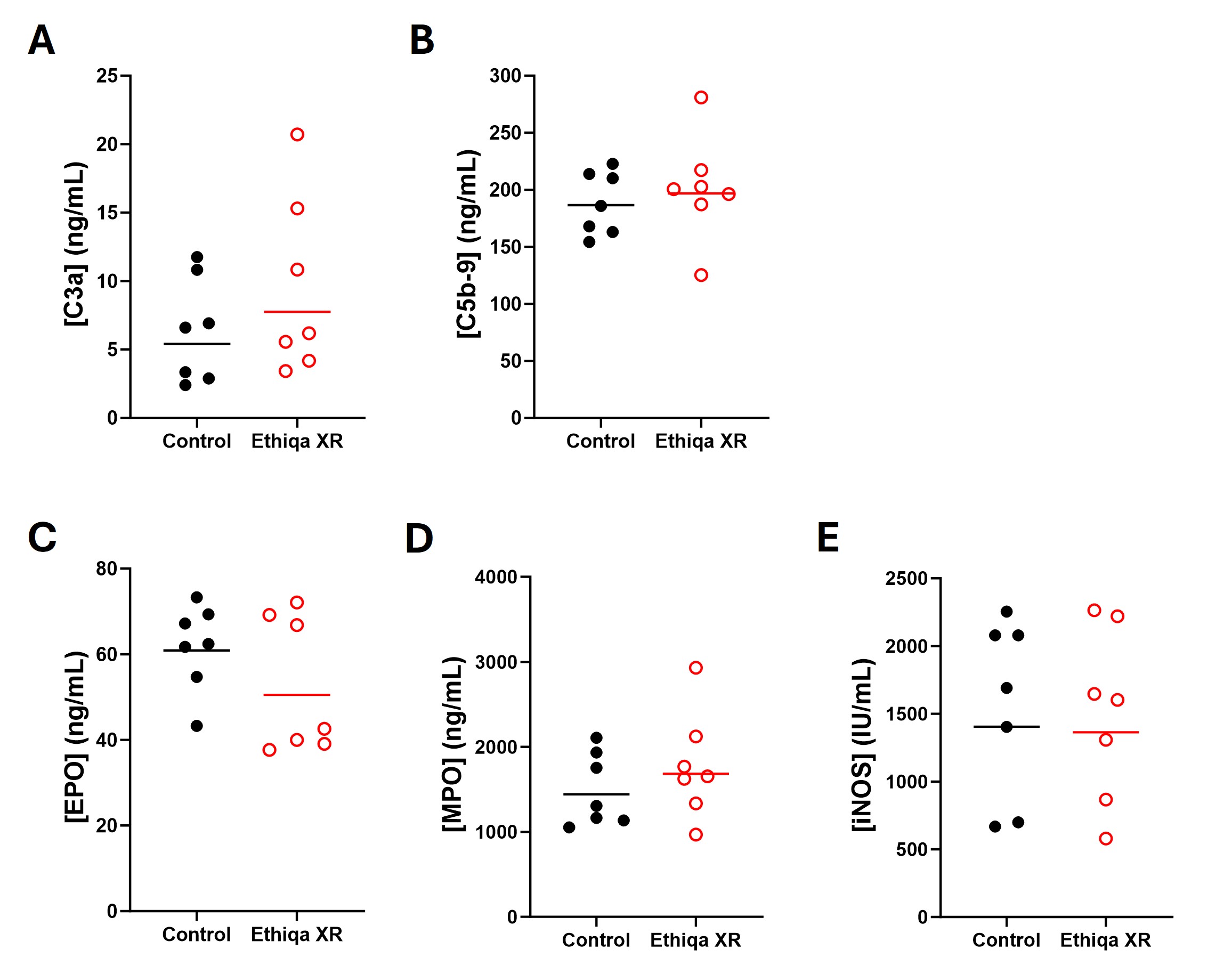

Supplement: Supplementary Figure 2 — ELISA analysis of complement, eosinophil, macrophage, and neutrophil activity of control or Ethiqa XR-treated BALB/c mice 24 h post-challenge with Y. pestis CO92. Scatter plots show the serum (n = 7 per group) concentration of (A) C3a and (B) C5b-9 as a measure of complement activation. Scatter plots (C-E) show the analysis of eosinophil, macrophage, and neutrophil activity by measuring the concentration of (C) eosinophil peroxidase (EPO), (D) myeloxidase (MPO), and (E) inducible nitric oxide synthase (iNOS) from the lung homogenates (n = 7 per group). Horizontal bars show the geometric mean. [file Image2.jpeg]

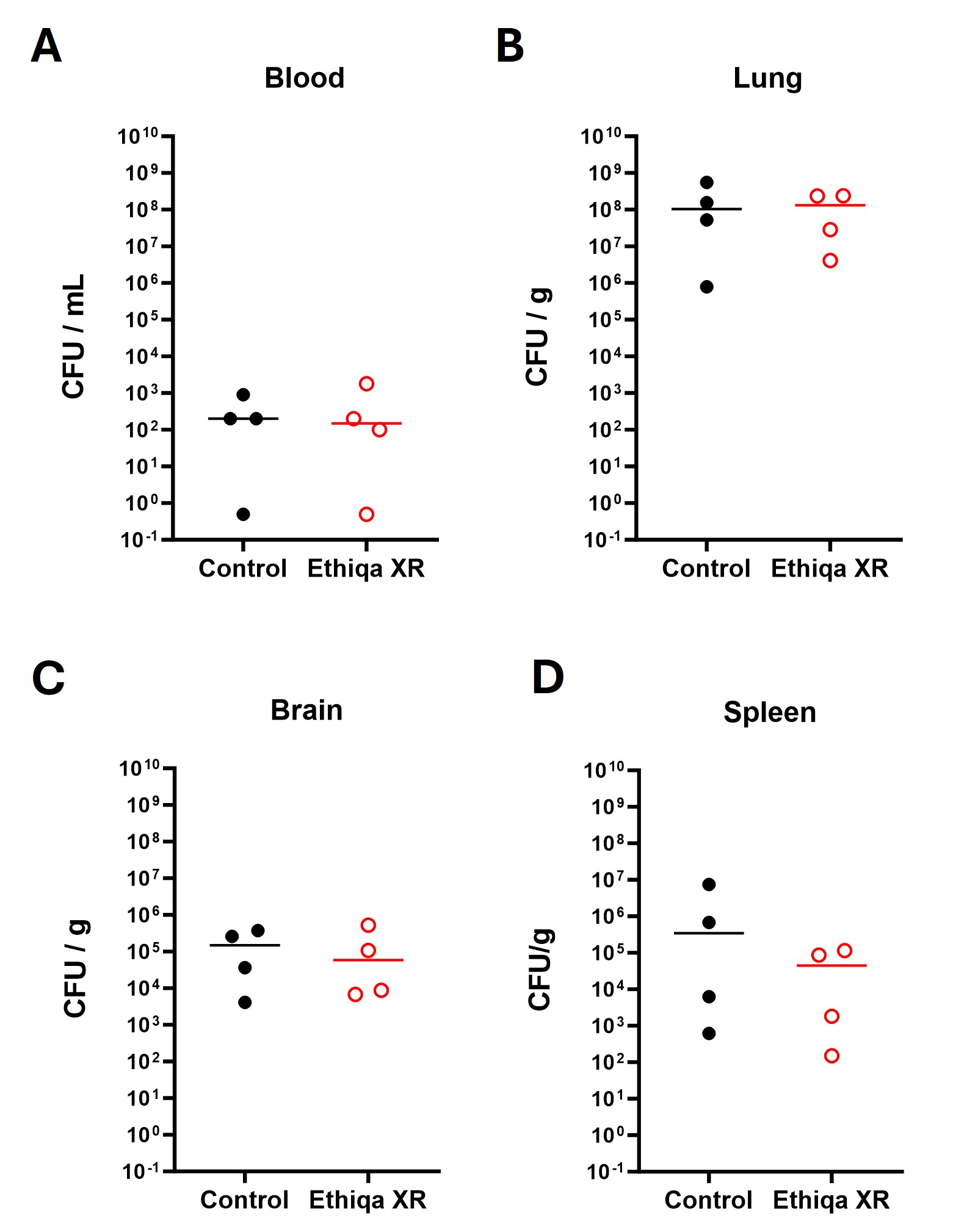

Supplement: Supplementary Figure 3 — Assessment of bacterial burden of Ethiqa XR-treated and control BALB/c mice 72 h post-challenge with B. pseudomallei K96243. Scatter plots (A–D) show the bacterial burden of BALB/c mice in (A) blood (B) lung, (C) brain, and (D) spleen homogenates (n = 5 per group). Scatter plots (E-H) show the bacterial burden in (E) blood, (F) lung, (G) brain, and (H) spleen homogenates (n = 4 per group). Horizontal line shows the median. [file Image3.jpeg]

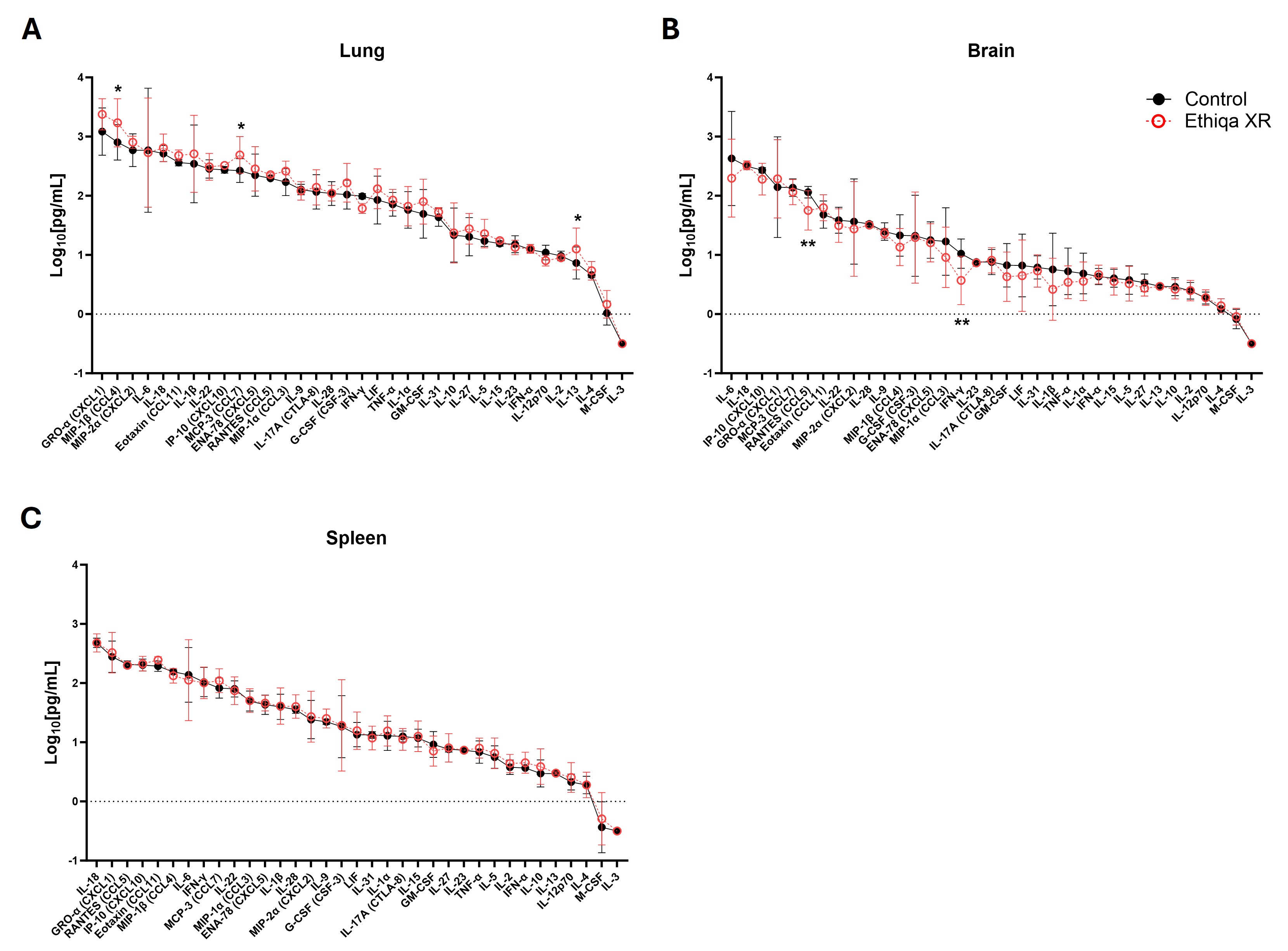

Supplement: Supplementary Figure 4 — Luminex analysis of the cytokine response from Ethiqa XR-treated and control BALB/c mice 72 h after B. pseudomallei K96243 challenge. Graphs show cytokine multiplex analysis in (A) lung, (B) brain, and (C) spleen homogenates (n = 4 per group). Data (mean ± SD) show log-transformed concentration values. Pairwise treatment groups were compared by linear mixed effects model with no multiplicity adjustment applied. Statistically significant changes are marked as: * (p ≤ 0.05), ** (p ≤ 0.005). [file Image4.jpeg]

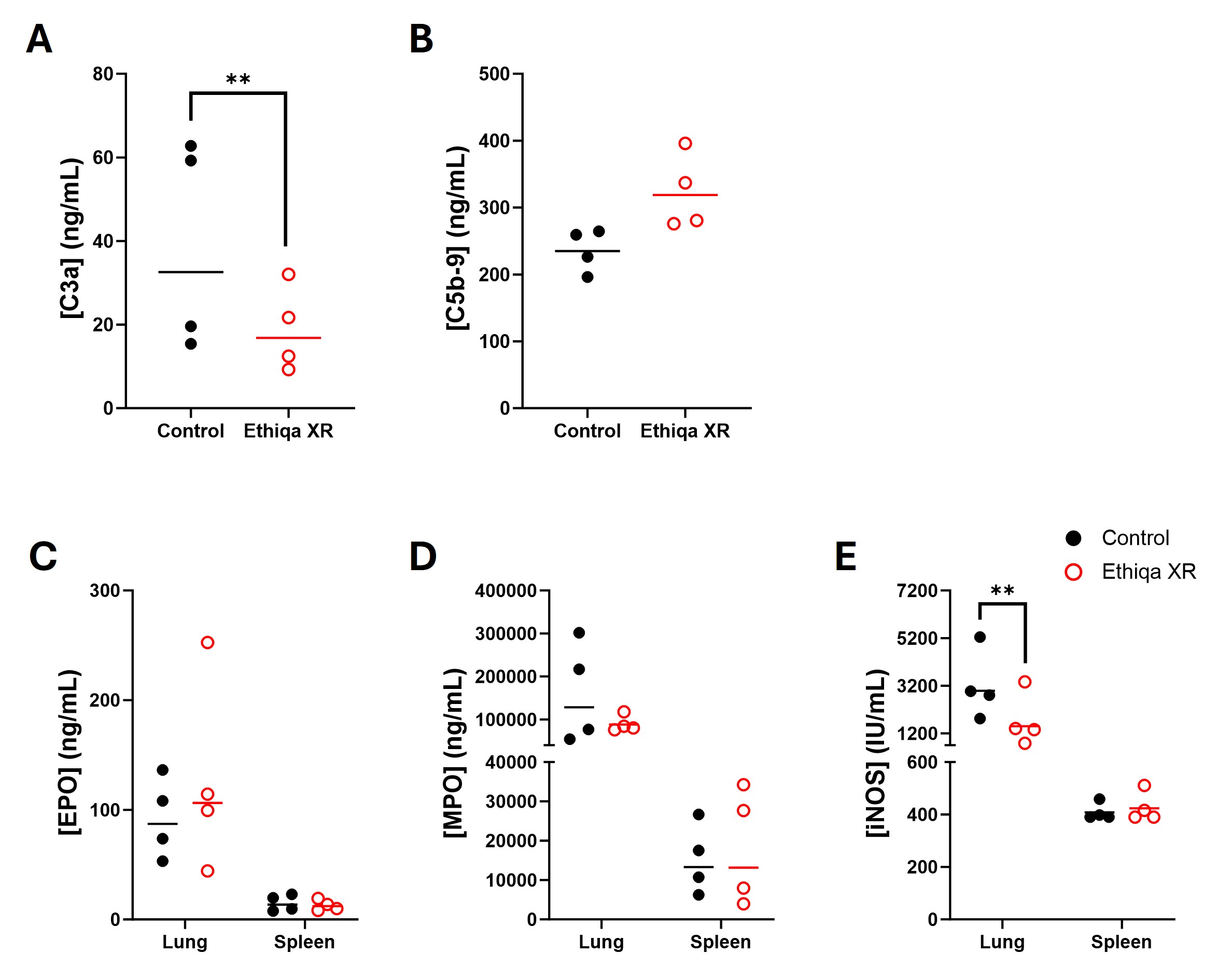

Supplement: Supplementary Figure 5 — ELISA analysis of complement, eosinophil, macrophage, and neutrophil activity of Ethiqa XR-treated and control BALB/c mice 72 h after B. pseudomallei K96243 challenge. Scatter plots (A, B) show complement activation measured as the serum (n = 4 per group) concentration of (A) C3a and (B) C5b-9. Scatter plots (C, E) show the analysis of eosinophil, macrophage, and neutrophil activity by measuring the concentration of (C) eosinophil peroxidase (EPO), (D) myeloxidase (MPO), and (E) inducible nitric oxide synthase (iNOS) from the specified tissue (n = 4 per group) homogenates. Horizontal bars show the geometric mean. Pairwise treatment groups were compared by linear mixed effects model. Statistically significant changes are marked as: ** (p ≤ 0.005). [file Image5.jpeg]
